# Epitaxially Driven Phase Selectivity of Sn in Hybrid Quantum Nanowires

**DOI:** 10.1021/acsnano.3c02733

**Published:** 2023-06-15

**Authors:** Sabbir A. Khan, Sara Martí-Sánchez, Dags Olsteins, Charalampos Lampadaris, Damon James Carrad, Yu Liu, Judith Quiñones, Maria Chiara Spadaro, Thomas Sand Jespersen, Peter Krogstrup, Jordi Arbiol

**Affiliations:** † Center for Quantum Devices, Niels Bohr Institute, University of Copenhagen, 2100 Copenhagen, Denmark; ‡ Danish Fundamental Metrology, Kogle Alle 5, 2970 Ho̷rsholm, Denmark; ¶ Catalan Institute of Nanoscience and Nanotechnology (ICN2), CSIC and BIST, Campus UAB, Bellaterra, 08193 Barcelona, Catalonia, Spain; § Department of Energy Conversion and Storage, Technical University of Denmark, Fysikvej, Building, Lyngby, 310, 2800 Denmark; ∥ NNF Quantum Computing Programme, Niels Bohr Institute, University of Copenhagen, 2100 Copenhagen, Denmark; ⊥ ICREA, Pg. Lluís Companys 23, 08010 Barcelona, Catalonia, Spain

**Keywords:** nanowires, topological materials, semiconductor-superconductor hybrid, Sn, quantum computing, interface, epitaxy

## Abstract

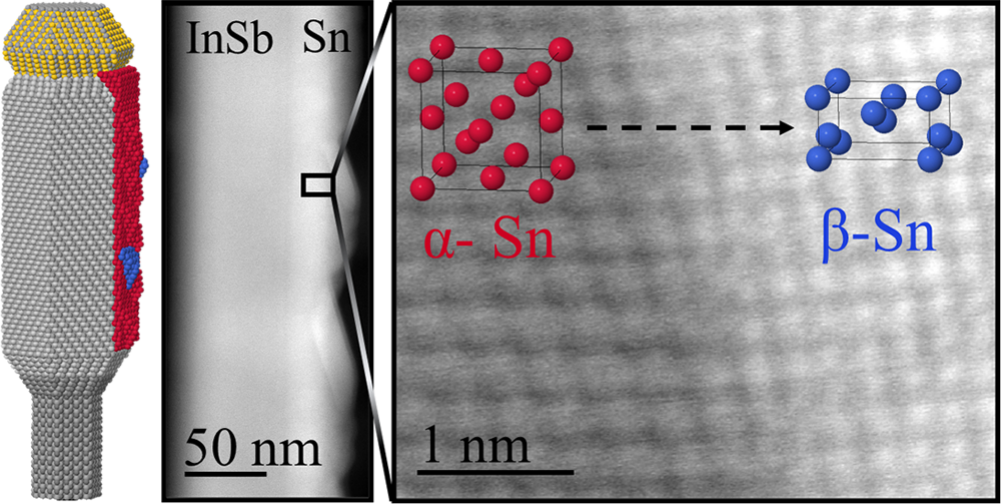

Hybrid semiconductor–superconductor nanowires constitute a pervasive platform for studying gate-tunable superconductivity and the emergence of topological behavior. Their low dimensionality and crystal structure flexibility facilitate unique heterostructure growth and efficient material optimization, crucial prerequisites for accurately constructing complex multicomponent quantum materials. Here, we present an extensive study of Sn growth on InSb, InAsSb, and InAs nanowires and demonstrate how the crystal structure of the nanowires drives the formation of either semimetallic α-Sn or superconducting β-Sn. For InAs nanowires, we observe phase-pure superconducting β-Sn shells. However, for InSb and InAsSb nanowires, an initial epitaxial α-Sn phase evolves into a polycrystalline shell of coexisting α and β phases, where the β/α volume ratio increases with Sn shell thickness. Whether these nanowires exhibit superconductivity or not critically relies on the β-Sn content. Therefore, this work provides key insights into Sn phases on a variety of semiconductors with consequences for the yield of superconducting hybrids suitable for generating topological systems.

Tin is a common group IV element used in a broad range of industrial applications including electronic circuits, optoelectronic devices, energy storage devices, and coating for commercial products.^1−7^ Recently, interest was further triggered with the promise of exploring exotic topological phases with Sn–semiconductor hybrids.^8−11^ However, the type of topology depends on which of the two major allotropes (α-Sn or β-Sn) is present. Hybridizing the semimetallic, cubic α-Sn phase,^6,12−18,20^ with semiconductors such as InSb (111) or (100) induces lattice mismatch-related strain fields. The resulting broken cubic symmetry can lead to topological insulator behavior.^8,9,14,21−26^ Conversely, metallic β-Sn is a comparatively dense body-centered tetragonal structure and exhibits superconductivity with a bulk critical temperature of 3.7 K.^6,12−18,20^ Hence, hybrids of β-Sn and one-dimensional semiconductors with strong spin–orbit interaction, such as InSb or InAs, may exhibit topological superconductivity in the presence of magnetic fields.^27−38^

Unleashing the potential of either of the two Sn phases—and excluding the potential for unwanted behaviors—requires phase-pure crystal growth, which is a challenge in thin films.^17,39−41^ In particular, the presence of α-Sn significantly reduces the yield of superconducting devices,^17^ and phase-mixed materials present problems with regard to fabrication. While research has been undertaken on crystal phase stability in bulk Sn,^6,16,17,19,21,39−46^ a comprehensive study of nanoscale Sn phase formation on semiconductor nanowires (NWs) is yet to be performed. Such knowledge is crucial to determining selective phase growth and controlling the interface properties of the Sn-based hybrid quantum heterostructures.

In this paper, we perform Sn thin-film growth on different semiconductor NWs and present an in-depth analysis. We tune the growth parameters to determine the optimal conditions and investigate the associated structural changes in the Sn film. When grown on B-polar cubic InSb and InAsSb NWs,^11,47,48^ Sn grows predominantly in the lattice-matched α phase. With increasing film thickness, we observe an increasing density of embedded β-phase grains. Based on the comprehensive crystal analysis of the growth evolution, we suggest a nucleation mechanism for β-Sn embedded within an α-Sn matrix. Obtaining hybrids with phase-pure β-Sn was possible by using InAs semiconductor NWs. Here, the β-Sn creates crystal domain matched grains, while differences in crystal symmetry between InAs and α-Sn and the large associated plane mismatches strongly suppress α-Sn nucleation. Finally, we correlate structural properties with electrical behavior at low temperatures. We show a strong correlation between the presence of superconductivity in ZB (InSb/InAsSb)-Sn with the β grain density and that those films expected to have a vanishingly low β-Sn content exhibit no trace of superconductivity. Conversely, β-dominant WZ(InAs)–Sn-based hybrids exhibit an induced superconducting gap with energy Δ = 440 μeV.

## Results and Discussion

### Multiphase Sn Crystals in Hybrid NWs

**Figure 1:**
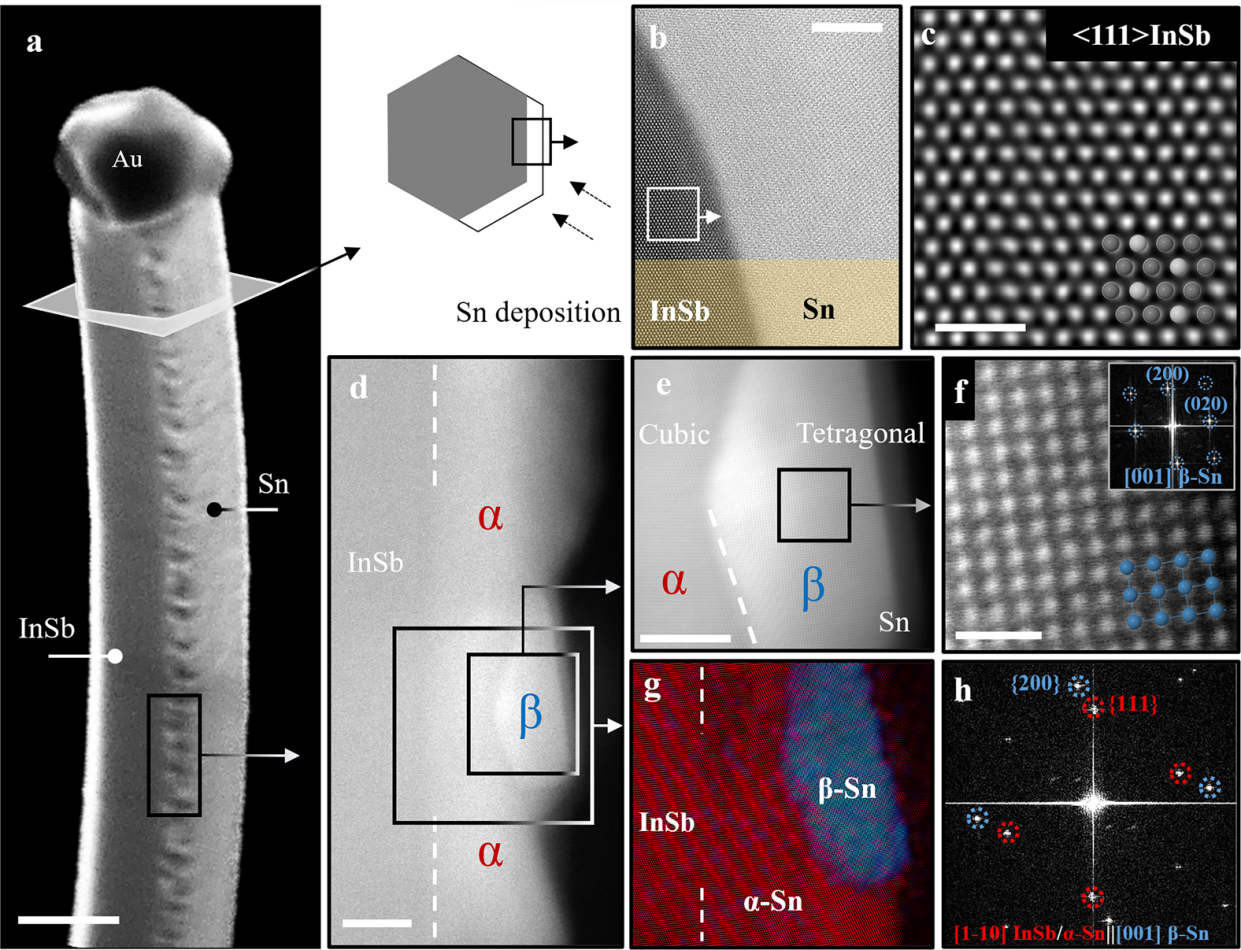
Hybrid InSb–Sn nanowires with multiple Sn phases. (a) Scanning electron microscopy (SEM) image of an MBE-grown InSb nanowire *in situ* hybridized with a Sn shell. (b) Cross-sectional aberration-corrected HAADF-STEM micrograph of the InSb–Sn interface. (c) Magnified HAADF-STEM image from the InSb segment showing cubic zinc blende crystal structure. (d) Low-magnification longitudinal HAADF-STEM micrograph of the heterostructure where coexistence of both α-Sn and β-Sn is observed. Dashed line indicates the starting of the Sn shell. (e) Magnified image of the interface between cubic-α and tetragonal-β phases. The white dashed line marks a {111}α-Sn plane interfacing with a β-Sn domain. (f) Zoom-in of β-Sn. (g) Fourier filtered map of crystal planes from the squared region marked in panel (d). Blue section matches the β-Sn phase, while red corresponds to InSb and α phases, which could not be unambiguously decoupled due to the equivalent cubic symmetries and their lattice mismatch being close to 0%. (h) Fast Fourier transform employed for (g) structural map showing indistinguishable plane reflections for InSb and α-Sn (red circles), while blue circles correspond to {200} planes of [001]-oriented β-Sn. Scale bars are (a) 100 nm, (b) 5 nm, (c) 1 nm, (d) 20 nm, (e) 10 nm, and (f) 1 nm.

Au catalyst-assisted NWs were grown on InAs (111)B or InAs (100)^11^ substrates using a molecular beam epitaxy (MBE) system. Similar to our earlier works,^11,49−51^ InSb and ternary InAsSb NWs were grown from InAs stems. Once the NWs were grown, Sn deposition was performed in a high-vacuum physical vapor deposition (PVD) chamber, which is *in vacuo* connected to the MBE (see Methods for details). Figure 1(a) shows a scanning electron microscopy (SEM) image of an InSb NW hybridized with an approximately 20 nm Sn shell. Here, Sn was grown on the selected facets of the hexagonal NW as indicated in the image. The crystal parameters and the elemental distribution mapping of these NWs are shown in Supporting Information, S1 and S2.

For crystal analysis, aberration-corrected high angular annular dark field (HAADF)–scanning transmission electron microscopy (STEM) micrographs were acquired in a cross-section of the hybrid NWs (Figure 1(b)). Clear contrast between the InSb core (dark) and Sn shell (bright) can be observed. The InSb segment can be identified as a cubic zinc blende (ZB) crystal with a lattice constant of 6.479 Å (see magnified HAADF-STEM micrograph in Figure 1(c)). In Figure 1(b), Sn seems to form a single crystal, but unambiguous phase identification was not possible due to either the α–β overlap or elemental migration and damage induced during focused ion beam (FIB)-assisted cross-section preparation (see Supporting Information S3). Therefore, we performed structural analysis on longitudinal views of NWs directly transferred to Cu grids by employing a combination of high-resolution TEM (HRTEM) and aberration-corrected HAADF-STEM imaging (see Figure 1(d)). Here three different HAADF intensities can be observed: the darker contrast originated from the InSb core, and the deposited Sn shows the light contrast with an enclosed area in the shell. Taking a closer look at Figure 1(d), we can observe that most of the shell matches the α-Sn phase, which is also face-centered cubic with a lattice constant of 6.489 Å. The small lattice mismatch (ϵ ∼ 0.15%) between ZB-InSb and α-Sn favors its growth on the NW interface, which we will elaborate on in a later discussion. The brightest small domain can be identified as a β-Sn grain embedded in the α-Sn matrix. From this and other analyzed NWs (see Supporting Information S4), we observed that β grains are mostly not grown directly interfacing with the InSb NW core, but they seem to appear on the epitaxial cubic α-Sn shell. The β grains typically show well-defined {020} faceting and seem to arise from {111} faceting on the α shell (indicated with white lines in Figure 1(e)). An atomic-resolution [001]-oriented β phase arrangement and its diffraction pattern are shown in Figure 1(f). Further, in Figure 1(g) we present a Fourier plane filtered phase map from the atomically resolved micrograph of the hybrid NW to highlight the multiphase structure. As we can see, due to the quasi identical atomic arrangement and atomic number, the InSb core and α-Sn have a perfect epitaxy and are barely distinguishable. On the other hand, due to the different crystal structure and reduced unit cell volume, the β-Sn grain shows distinct contrast and exhibits a sharp interface with the other cubic structures. These differences in the lattice arrangement can be noticed in the fast Fourier transform (FFT) displayed in Figure 1(h).

### Optimal Conditions for the Sn Shell on NWs

Thin-film formation of Sn crucially depends on the substrate temperature and chemical potential of the incorporation sites, as they exponentially govern the adatom diffusion length on the NW facets.^11,52,53^ Low-temperature growth assists in minimizing the adatom diffusion length and drives the Sn film to wet on the NW surface. Besides, the chemical potential of the incorporation sites depends on the change in Gibbs free energy that is determined by the interface energy density between NW facets and deposited Sn.^11,52,53^ In the initial stage of Sn growth, the interface energy depends on the lattice mismatch between two materials and the area of the interface. In the latter stage, strain energy also plays a key role in defining the interface energy density and evolving grain boundaries. Hence, selection of NW and a Sn phase with low residual mismatch will ease the formulation of a continuous Sn shell. In contrast, if the NW and Sn phases have a high residual mismatch, then a significant thermodynamic force will require to make a continuous film on NW facets. Further, a high flux rate also assists Sn shell growth by increasing the adatom concentration on the NW facets. High adatom concentration increases the nucleation density on the given surface, which also decreases the average adatom diffusion length. In a nutshell, a combination of the low substrate temperature, favorable interface energy density, and high flux rate can provide an optimized Sn shell on the NW facets. Hence, we maintained the Sn flux rate in the range of 3–5 Å/s, and the sample holder temperature was either approximately −100 or −150 °C during the growth. A detailed study of growth temperature effect on the hybrid Sn shell structure is presented in the Supporting Information, S5–S9.

### β-Sn Nucleation in an α-Sn Matrix

**Figure 2:**
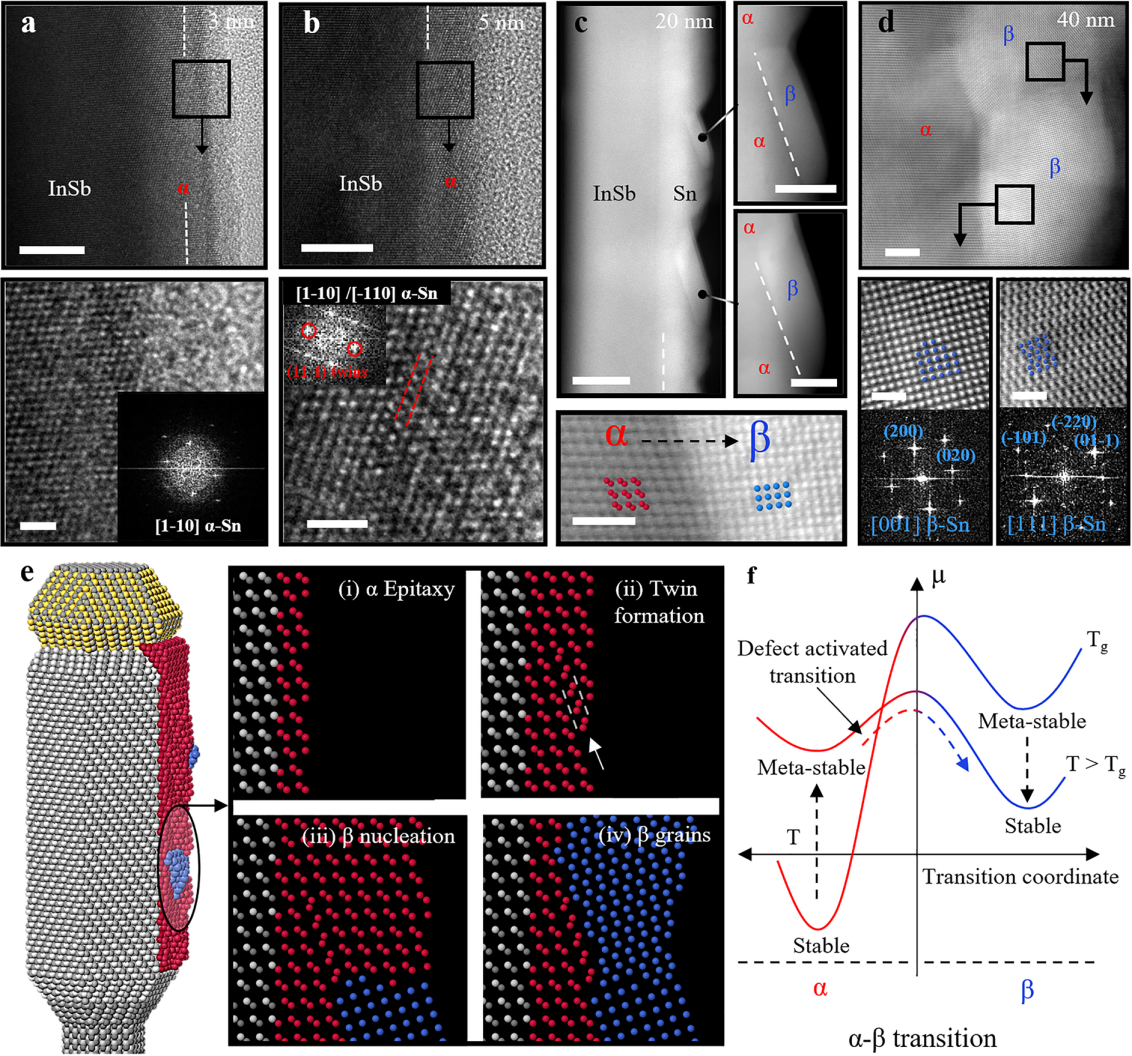
Sn shell evolution and β-Sn grain formation. (a) HRTEM micrograph of the InSb-Sn NW, where approximately 3 nm Sn was deposited. Due to the ultrathin film and epitaxial relation, the interface is just distinguished by a subtle contrast variation. FFT on a magnified image of the interface confirms the α-Sn phase. (b) HRTEM image of the approximately 5 nm Sn shell where an initial stage twin plane is identified. (c) Large-scale low-magnification HAADF-STEM micrograph of a 20 nm Sn shell where clear contrast between α-Sn and β-Sn can be observed. Zoom-in shows β-Sn grains with well-defined faceting that seem to arise from {111} faceting of the α-Sn shell (white dot lines). The bottom panel shows an atomically resolved HAADF-STEM micrograph of the α–β interface. (d) HAADF-STEM micrograph of 40 nm Sn deposited on InSb. β-Sn grains can be observed on top of the initial α-Sn. (e) Hypothesis for the α, β nucleation of the Sn film on the InSb NW. (i) Initially α-Sn grows epitaxially on the lattice-matched InSb cubic interface. (ii) A few layers away from the interface twin defects appear (indicated by the arrow) mostly oblique to the growth direction. (iii) These twin planes act as preferential nucleation centers for the β-Sn phases when the activation energy barrier is overcome and, hence, a β grain forms. (iv) For thick shells, large β-Sn grains with different orientation can be observed and initial {020}-β faceting dissolves. (f) Schematic of the α ↔ β transition as a function of chemical potential (μ) and temperature. Here, *T*_g_ (during growth) and *T* are the holder temperatures. Scale bars are (a) 10 nm and 2 nm, (b) 10 nm and 2 nm, (c) left: 50 nm, right: 20 nm (both), bottom: 2 nm, (d) top: 5 nm, bottom: 1 nm (both).

To understand the thin-film evolution, we performed an extensive structural study varying the Sn shell thickness (Figure 2(a–d)). In Figure 2(a), approximately 3 nm of Sn was deposited on an InSb NW. As α-Sn forms an epitaxy with InSb, it was difficult to differentiate the interface. However, a weak contrast fluctuation from the interface (indicated with the white dot line) can be observed. We did not see any tetragonal β-Sn domain in this extremely thin shell, which would have been noticeable on the cubic structure. More details can be found in Supporting Information S10. In Figure 2(b), approximately 5 nm of Sn was deposited on the InSb NWs. Here, a dominance of α-Sn is still observed, and some twinning planes start to be visible, as indicated in the magnified section and inset diffraction pattern. In addition, a few seed-stage β-Sn grain domains are also observed in this case (see Supporting Information S11). In some sporadic cases, we observed β-Sn grains close to the interface with InSb, but unambiguous resolution of possible direct contact between InSb and β-Sn could not be determined due to the 3D structure of the system. In any case, the threshold thickness for which we start to observe β-Sn nucleation is 5 nm; therefore, we assume some epitaxial α-Sn might be present prior to it even though it might not be the only β-Sn formation mechanism. In Figure 2(c), approximately 20 nm of Sn was deposited, and full-fledged faceted [001]-oriented β-Sn growing on the {11–1}α-Sn planes can be observed. In the magnified inset images, a clear preferential faceting of β-Sn on α can be observed, which belongs to the {020– β family of planes (see more examples in Supporting Information S4). At the bottom of Figure 2(c), we present an atomically resolved micrograph of the α–β interface between cubic and tetragonal Sn phases. Finally, an approximately 40 nm deposited Sn shell is shown in Figure 2(d). The shell exhibits a continuous but rough morphology (also see Supporting Information S12). Here all the NWs investigated by TEM showed a similar nanostructure, with multiple β-Sn grains being randomly oriented (most of them out-of-axis), and they are grown from the α phase. Figure 2(d) shows an example of two neighboring β-Sn grains oriented in two different zone axes, highlighting the polycrystalline nature of the β-Sn shell in contrast to the fully epitaxial α-Sn. It is also notable that the strong {020}-β faceting vanishes as the β grains grow. We also observe an increasing density of β-Sn grains presenting different orientations as we increase the shell thickness. Therefore, from a fully epitaxial α-Sn shell promoted by lattice-matched InSb templates, we observe an evolution to form a polycrystalline β-Sn shell in thick films.

Based on the experimental analysis, in Figure 2(e), we present a hypothetical model of the structural evolution of Sn phases in the shell grown on a cubic NW template. The chemical potential of the α-Sn or β-Sn phase on the NW can be associated with the chemical potential of the reference bulk phase (μ_*i*,bulk_) and thin film (μ_*i*,ex_). The chemical potential in the thin film will again depend on the NW surface (μ_*i*,surf_), interface (μ_*i*,int_), strain (μ_*i*,strain_), and grain boundary (μ_*i*,gb_). Hence, α or β crystal formation is an accumulated factor of all those parameters:
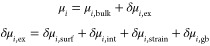
where *i* = α, β.

Since bulk α-Sn is stable below 13.2 °C and the interface energy between InSb and α-Sn is very small due to its close lattice matching, in our low-temperature growth condition, it is favorable to form α-Sn in the interface. Hence, the epitaxial Sn film appears initially (Figure 2(e)(i)). In the later stage (Figure 2(e)(ii)), twin formation in the α-Sn shell initiates mostly in the {11–1} plane away from the interface. Such twins are oblique to the growth directions and can be found all over the shell, as shown in the Supporting Information S5–S9. Although epitaxial α-Sn is grown at the interface, it is metastable in temperature exposure and can change the crystallinity if the chemical potential of α-Sn overcomes the kinetic energy barrier between α and β phases. The schematic of activation energy dependent α ↔ β transition is shown in Figure 2(f) and can be expressed as follows:
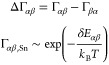
Here, ΔΓ_*αβ*_ is the growth rate and *δE*_*αβ*_ is the activation energy, which is dependent on μ_*i*_. Next, tetragonal β grains nucleate on top of the cubic α-Sn shell, overcoming the activation energy barrier. This activation energy barrier may be overcome due to the change in local conditions during the growth, causing a transition from the α to β phase and *vice versa*. However, a likely possibility is that β nucleation occurs while the sample is abruptly exposed to a temperature that is higher than the growth temperature (see Figure 2(f)) (perhaps right after the low-*T* Sn growth). As we observed, these β-Sn grains typically display well-defined faceting that seems to arise from {111} faceting on the α-Sn (Figure 2(e)(iii)). Hence, it can be inferred that β-Sn overgrows from an α structure by nucleating in twin-induced rough sites instead of a flat surface of InSb NW. Finally, for a thick Sn film (Figure 2(e)(iv)), β-Sn grains gain volume and often multiple grains coalesce into one large grain; simultaneously more β grains start to nucleate in different places of the shell. As a result, different orientations of β-Sn grains can be observed. Once both crystal phases are stabilized at room temperature, no further transition between α-Sn and β-Sn happens, as the activation barrier for the transition is very high at this point. Hence, both α and β crystal phases coexist in the Sn shell on cubic NWs. In Supporting Information S13 we performed thermal annealing on the 20 nm shell NWs and observed no changes in the Sn heterostructure even when turning the conditions to completely favor the β-Sn phase, proving the structural stability of the achieved configuration.

### Increasing Interfacial Lattice Mismatch

Based on the above discussion, it can be presumed that the lattice parameter of the NW core is key to control the Sn-shell phase formation in the hybrid. Therefore, other NW compositions were employed to decrease the lattice match of α-Sn and, hence, hinder its formation. With this purpose, we grew NWs with 80% Sb and 20% As composition, which implies a lower lattice constant than in pristine InSb NWs given Vegard’s law,^54^ while preserving the ZB cubic phase. This configuration implies an increase in lattice mismatch between cubic α-Sn and the NWs, rising from 0.15% with pure InSb to 1.5% for InAs_0.2_Sb_0.8_.

**Figure 3:**
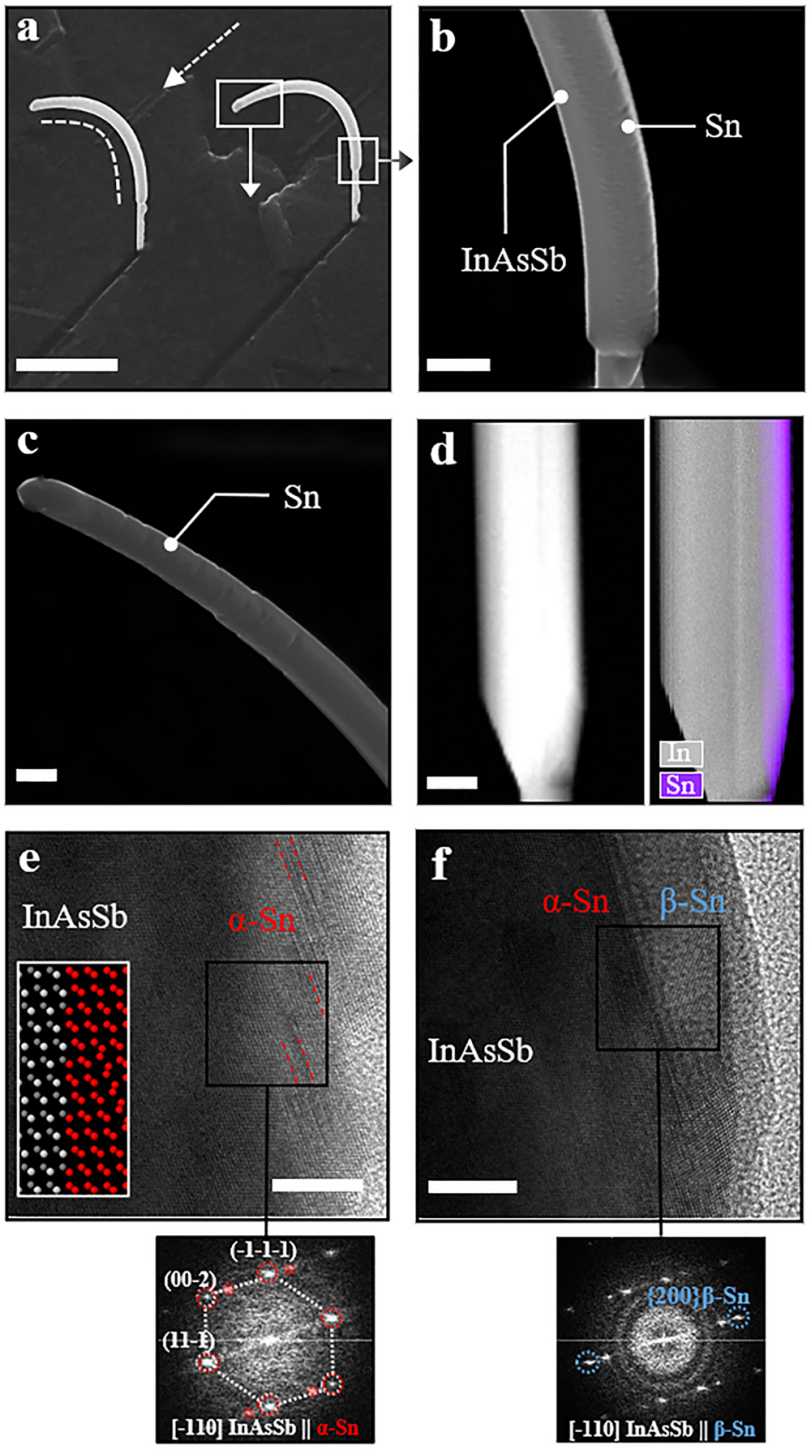
Morphology and structural analysis of InAsSb/Sn NWs. (a) Tilted SEM image of the InAsSb/Sn NWs. Here 20 nm Sn is grown on the three facets of InAsSb NWs. The white arrow shows the deposition direction of the Sn. (b) Magnified bottom section of the NW from panel (a). Continuous but rough morphology of the Sn shell can be observed. (c) Magnified top section of the NW from panel (a). (d) HAADF-STEM image of InAsSb (left) and EELS-based In/Sn compositional map, which reveals continuous Sn coverage. (e) HRTEM image and its FFT of α-Sn on InAsSb. The interface is barely distinguishable due to the same crystal symmetry (see inset model) between cubic α-Sn and ZB InAsSb, but twinning is present in Sn. (f) HRTEM image and its FFT of observed β-Sn grain on α-Sn on InAsSb. Scale bars are (a) 1 μm, (b–d) 100 nm, and (e, f) 20 nm.

Figure 3(a) presents InAsSb NWs with a 20 nm Sn shell grown on selected facets of the NWs, where the growth rate was approximately 3 Å/s and the sample holder temperature was approximately −150 °C. Magnified sections of these NWs are shown in panel (b) (bottom part) and in panel (c) (top part). Two major observations from these hybrids are, first, the Sn shell exhibits a continuous but inhomogeneous morphology on InAsSb NWs compared to InSb NWs; second, the hybrid InAsSb–Sn NWs show strong bending along the deposition direction. Figure 3(d) presents a low-magnified HAADF-STEM image and an electron energy loss spectroscopy (EELS) elemental map of In/Sn in an NW, which confirms the continuity of the shell. Taking a close look into the crystal arrangement of the shell, Figure 3(e) shows an example of the most commonly observed crystal phase of Sn on the InAsSb NWs, belonging to the α-Sn phase and forming a similar structural configuration to that observed in InSb NWs. As previously discussed, the α-Sn shell contains several planar defects including twinning and stacking faults, which form at ∼71° with the growth direction plane. The model in the inset of Figure 3(e) shows the atomic arrangement between α-Sn and InAs_0.2_Sb_0.8_. In addition, we could observe some β-Sn grains embedded in the α-Sn, also exhibiting the same crystal configuration with well-defined faceting in the {200} planes, but they are discretely distributed through the NW shell, which displays a prevalence of the α phase. More micrographs on different areas are presented in the Supporting Information (S14 and S15).

Hence, despite increasing the interfacial lattice mismatch of the NW with the cubic Sn phase, we still obtain a preference for α-Sn nucleation. We presume that the increase in interface energy results in compressing strain fields that relax toward the outer Sn shell and thus strongly bend the NW (Figure 3(a)). A schematic of compressive strain fields originating the bending is displayed in Supporting Information S14. As a consequence, this configuration still favors α-Sn nucleation over β-Sn. In addition, this bending can be responsible for the rough morphology observed in the shell. Although the Sn crystal structure on InAsSb is identical to the InSb one, careful attention should be put on the resulting electronic behavior of the InAsSb–Sn system because of the strain accumulation at the interface, as it is capable of locally modifying its properties.^55^

### Selective β-Sn Nucleation in a Poor Lattice Matched WZ Core

**Figure 4:**
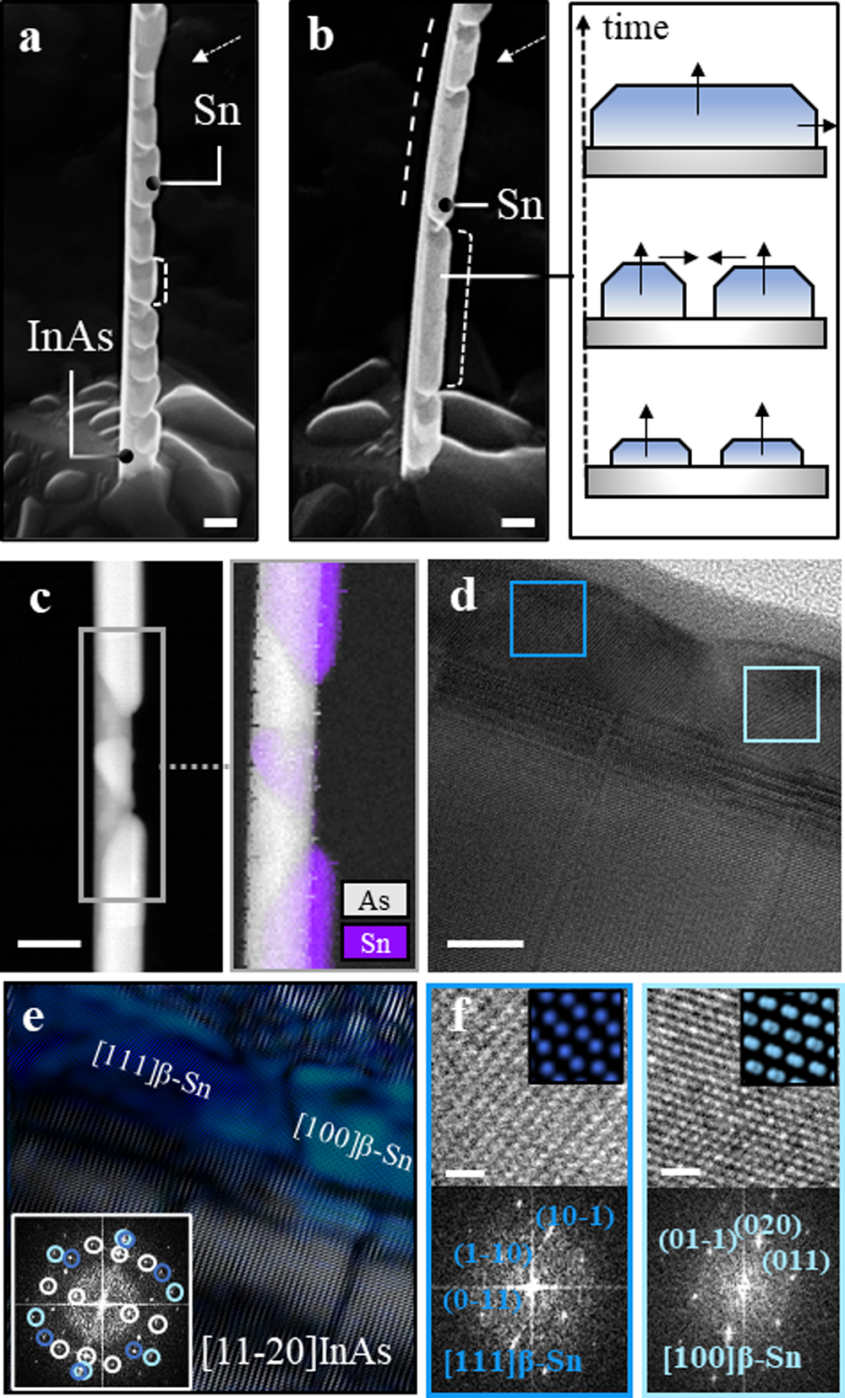
Sn shell growth on wurtzite InAs NWs. (a) Tilted SEM image of the InAs NW with a Sn shell. Sn forms discrete grains with uniform thickness (∼22 nm), but nonuniform length (>100 nm). Arrow shows the deposition direction. (b) Doubling the Sn deposition time, where the Sn shell thickness is ∼45 nm. With longer growth time nearby, Sn grains grow larger and eventually collapse on each other as shown in the right-side schematic. (c) HAADF-STEM micrograph of an InAs-Sn NW and EELS compositional mapping of As and Sn on the squared region. (d) HRTEM micrograph showing two Sn grains in direct contact with the InAs NW. (e) Fourier filtered structural map of (d), showing the spatial distribution of two differently oriented β-Sn grains on InAs. (f) Zoom-in of the squared regions in (d) with their corresponding indexed FFTs and phase identification as β-Sn. Scale bars are (a–c) 100 nm, (d) 10 nm, and (f) 1 nm (both).

Finally, we investigate the deposition of Sn on the WZ InAs NWs. Figure 4(a) shows InAs NWs with a ∼20 nm Sn shell grown at the rate of 3 Å/s and a holder temperature of approximately −150 °C. Sn forms discrete grains with uniform thickness on InAs NW facets. The NW crystallizing in the hexagonal phase implies that there is no particularly favorable lattice-matched configuration for either of the two Sn phases, leading to a high interface energy between these materials (see Supporting Information S1).^11,53^ As a result, a high thermodynamic driving force is needed for Sn to wet on the InAs NW facets during the growth. Hence, in our growth conditions with a limited low-temperature range, Sn remains as dewetted islands on the InAs NW. Therefore, to achieve a continuous film with the same thickness, the temperature should be further lowered so that the Sn adatoms can be immobilized during the growth. Another way to achieve a continuous film is by increasing the Sn thickness. Figure 4(b) shows an example of ∼45 nm Sn deposited on an InAs NW keeping the same growth conditions. Doubling the Sn thickness enhanced the length of the islands. However, the shell remains discrete and more material should be deposited to achieve a continuous film. As presented in the schematics on the left, upon reaching a critical thickness, the neighboring islands coalesce and evolve to form large islands, which can merge again with the adjacent ones. Figure 4 (c) shows an EELS-based compositional map of Sn and As of an NW together with a HAADF-STEM micrograph of the area of study. We can observe that despite the grains tending to be relatively flat and homogeneous in terms of thickness, the grain size distribution is highly inhomogeneous. When moving to analyze the crystal phase of Sn, we found most of the grains, including the larger ones, are out-of-axis with respect to the NW (Supporting Information S16), while we could find some small sections being in-axis with the InAs (see Figure 4(d–f)). We can identify all these grains as β-Sn (Figure 4(f), and they are in direct contact with the InAs NW (Figure 4(e)), with no presence of α-Sn prior to their nucleation. More micrographs with β phase identification are available in the Supporting Information (S16–S20). Based on the analyzed structures, we presume that the presence of a hexagonal crystal as growth template inhibits the formation of the cubic α-Sn phase. In the previous cases, the same cubic crystal nature of InSb and InAsSb acted as a template for cubic α-Sn growth and made it the most stable phase by hindering the nucleation of tetragonal β-Sn. However, when employing hexagonal InAs as a core material, the crystal symmetry equivalence is broken. The most similar configuration between cubic and hexagonal would be InAs(0002)/α-Sn(111),^56,57^ but this still implies a 7% plane mismatch along the NW growth axis direction. Therefore, lower mismatch β-Sn growth gets favored instead of α-Sn, and it is the only phase we observed in all the NWs we examined. While in previous cases β-Sn grains nucleated on the α-Sn matrix showed some preferential out-of-plane orientation, β-Sn grains on InAs show a high degree of randomness regarding their orientation with respect to the InAs lattice, especially for smaller grains. However, for the long-range grains, we observe a dominant Sn orientation in the in-plane direction, although it is not in axis with the InAs crystal. In Supporting Information S16–S18 multiple HRTEM micrographs of the InAs-Sn heterostructure with larger Sn grains are presented. A 2.9 Å plane reflection parallel to InAs(0002) is visible in all of them and creates periodic moiré patterns in areas overlapping with InAs. This reflection belongs to the {020}β-Sn family of planes. Given the differences in lattice symmetry between β-Sn and WZ InAs, there is no low-index 3D configuration with low associated mismatch in all matching planes, and this explains the appearance of multiple crystal orientations in the heterostructures. In the case of the observed InAs(0002)/β-Sn{200}, they provide a mismatch of εInAs(0002)/β-Sn{200} = 16%. However, the domain formation consisting of 5×(002)InAs with 6×{200} β-Sn planes reduces the mismatch to ε5×InAs(0002)/6×β-Sn{200} = 0.017%. In fact, the moiré periodicity found in the overlapping areas matches with this plane combination (S16–S18). We assume that this low plane mismatch domain is therefore more energetically favorable than the other observed orientations and can be maintained in a longer spatial range, thus being the orientation observed in larger Sn grains. More details and schematics of the plane matching in the formed domains can be found in S16.

Last but not least, in order to cross-check that the observed preclusion of α-Sn growth on InAs NWs is caused by its structural symmetry rather than its composition, we radially grew an approximately 2 nm shell of InAs covering the InSb NW before depositing Sn (see Supporting Information S21). In these conditions, InAs achieves ZB phase and remains completely strained on top of the InSb, adopting its crystal symmetry and lattice constant. We observed that regardless of the presence of InAs covering InSb, Sn crystallizes mainly in the α phase, with some β grains being embedded on it (see S21). This fact is consistent with the NW crystal structure being one of the key parameters driving Sn phases on hybrid NWs. A schematic of Sn phase dynamics as a function of lattice parameters is presented in Supporting Information S22.

### Low-Temperature Electrical Measurements

**Figure 5:**
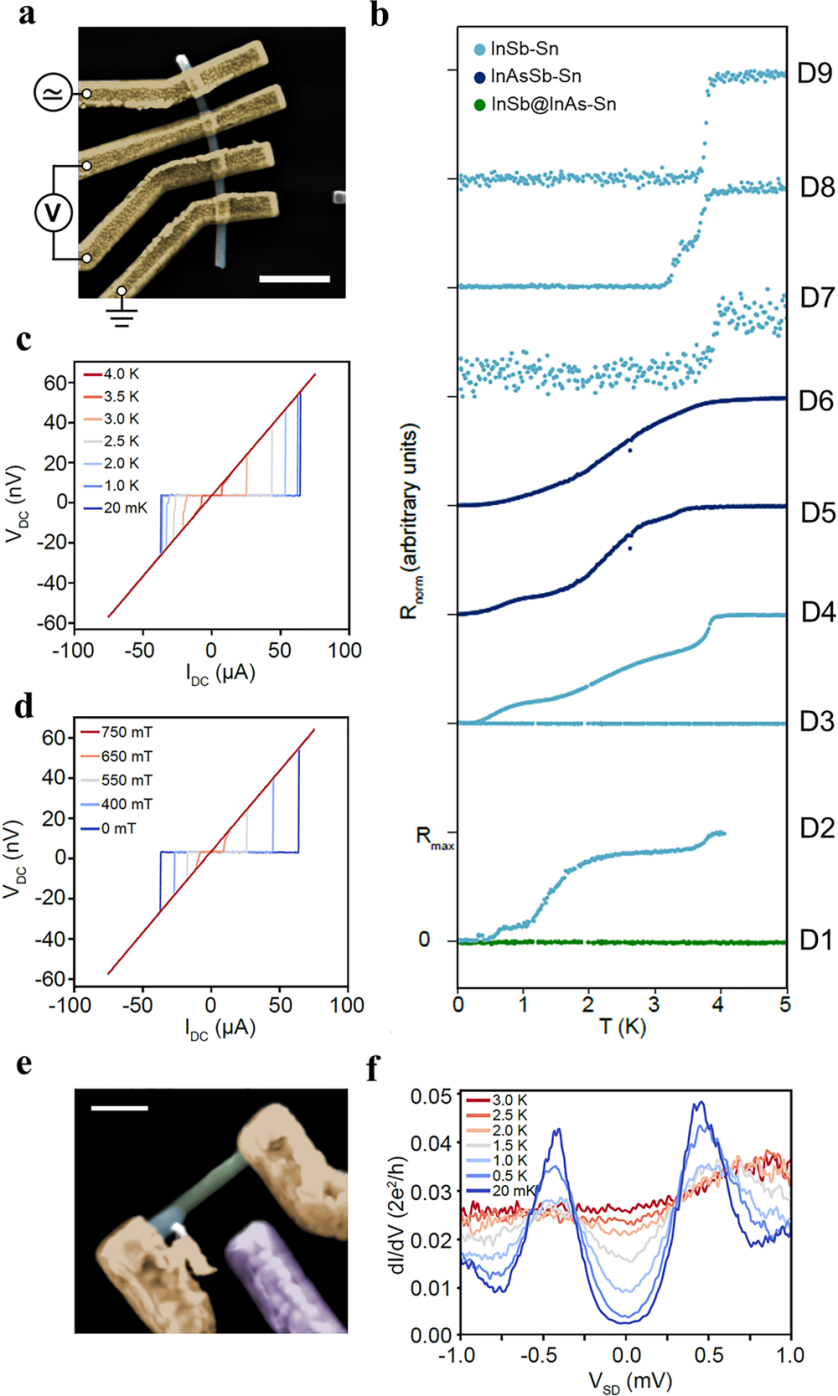
Low-temperature measurements of NW–Sn hybrid devices. (a) SEM micrograph of a typical device. (b) Temperature dependence of the four-terminal differential resistance for nine different devices from three different hybrid types. See the text and Supporting Information S23 for details. For each device the resistance is normalized to the value at the highest temperature and curves are offset for clarity. (c, d) *V–I* curves for device D9 measured as a function of temperature and perpendicular magnetic field, respectively. The sweep direction was from negative to positive current. (e) SEM micrograph of a two-terminal InAs–Sn N–S device. (f) Differential conductance as a function of voltage bias measured for the InAs–Sn device in different temperatures. Scale bars are (a) 1 μm and (e) 200 nm.

To investigate the superconducting properties of the hybrids, we fabricated and measured electrical devices at low temperatures. Individual hybrid NWs were transferred from the growth substrate to a substrate of highly doped silicon capped with 200 nm of SiO_2_. Nanowires were located with respect to predefined alignment grids, and Au/Ti contacts were defined using standard e-beam lithography and metal evaporation. See Methods section for details of the fabrication. Figure 5(a) shows a SEM micrograph of a typical device with four contacts on the same NW. A 10 nA ac current was sourced between the outer contacts, and the resulting voltage drop between the inner pair was measured using standard lock-in techniques, yielding the resistance between the inner pairs. Measurements were performed in a dilution cryostat with a base temperature of 15 mK. Figure 5 shows the temperature dependence of the resistance normalized to the value at the highest temperature for nine different NWs from six different growths of three different hybrid types. D2–D4 and D7–D9 are InSb-Sn devices, D5 and D6 are InAsSb-Sn devices, and D1 is an InSb(InAs shell)–Sn device. See the table in Supporting Information S23 for the growth parameter details. Devices D3 and D1 show no changes in the resistance upon cooling, which we attribute to the absence of β-Sn grains in the gap between the inner voltage probes for these devices. All other devices, however, show a clear drop in resistance around 3.7–3.9 K close to the expected transition temperature of β-Sn. While D9 shows an abrupt drop to an *R* = 0 Ω at ∼3.8 K, the remaining devices exhibit a gradual transition with multiple distinguishable steps upon cooling. This behavior is consistent with a Sn shell composed of discrete β-Sn grains electrically coupled through a non-superconducting α-Sn matrix and the semiconductor NW. At *T*_C_^β^, isolated β-Sn grains turn superconducting, and while the resistance drops, the resistance remains finite. Other grains have transition temperatures suppressed either due to finite size effects^58^ or because of an inverse proximity effect from the surrounding matrix. Upon lowering the temperature, a gradual transition thus occurs until a global superconducting phase is established possibly including proximity coupling neighboring grains through the normal matrix or semiconductor wire. A similar trend was shown in systems of designed Josephson arrays of superconducting regions in a normal matrix.^59^ These results are consistent with the structural analysis presented in Figures 1–3 for ZB InSb and InAsSb NWs based hybrids. Figure 5(c,d) show the *V–I* characteristics of D9 as a function of temperature and perpendicular field, respectively. The measurements were performed sweeping the current from negative to positive, and the transition from the resistive to the superconducting state upon sweeping *I* from a large value toward zero occurs at a smaller absolute current than the transition from the superconducting to the dissipative state observed when increasing *I* from zero. We attribute this asymmetry to Joule heating in the normal state. The critical current can be observed up to 3.5 K (for *B*_⊥_ = 0) and *B*_⊥_ = 650 mT (for *T* = 15 mK), consistent with panel (b).

Finally, to measure the size of the superconducting gap and confirm that superconductivity extends from the Sn shell into the semiconductor NW, we further fabricated a two-terminal device on the InAs–Sn stem, as shown in Figure 5(e). Here a large isolated grain could be seen with one contact positioned on the grain and the other on the adjacent Sn-free semiconductor segment. As explained in Figure 4, the discrete Sn grain here is expected to be only the superconducting β-Sn phase without the α mix. The electron density in the semiconductor can be tuned using the potential on a nearby electrostatic side-gate, and close to pinch-off a quantum dot (QD) forms in the semiconductor (see Supporting Information S24 for further characterization). In Coulomb blockade the QD acts as a tunnel barrier allowing tunnel spectroscopy of the density of states in the semiconductor. Figure 5(f) shows the measured differential conductance d*I*/d*V*_SD_ vs *V*_SD_ for different temperatures. Here *V*_SD_ is the applied voltage bias. Superconducting coherence peaks are observed at *V*_SD_ = ±Δ*/*e*, corresponding to an induced gap of Δ* = 440 μeV, close to the value expected for a gap induced by β-Sn.

## Conclusion

In summary, we have presented hybrid NWs using various III-V semiconductors and Sn shells and performed an in-depth analysis of Sn crystal phase formation in the heterostructure. InSb, InAsSb, and InAs NWs were grown using MBE and later *in situ* hybridized with Sn by determining optimal conditions and attaining the utmost interface quality. We have provided a comprehensive structural analysis at the atomic scale that reveals the coexistence of the two different Sn crystal phases (α-Sn and β-Sn) when deposited on cubic crystal NWs like InSb or InAsSb. Based on the experimental assessment in different stages of the Sn film, we proposed a hypothesis of α,β-Sn phase nucleation in the hybrid. The NWs with a cubic lattice provide a template to epitaxially grow α-Sn due to their quasi-identical crystal structure (space group, atomic positions, and lattice constant). Later, β-Sn nucleates preferentially from the defective sites of the film by overcoming an activation energy, and eventually isolated grains are observed to be embedded in the dominant α-Sn phase. The density of β-Sn grains has been shown to increase with the shell thickness. On the contrary, selective growth of β-Sn was achieved on InAs NWs, which is assumed to be a result of the poor lattice match between the WZ hexagonal crystal and the cubic α phase. In agreement with the structural analysis, low-temperature electrical measurements on InSb–Sn and InAsSb–Sn devices manifest the influence of discrete β-Sn grains on the surrounding α-Sn matrix. While one device demonstrated a sharp SU transition, others show a multistep transition, which is possibly due to the inverse proximity effect from a conducting–nonconducting mixed structure. Additionally, by measuring a phase-pure β-Sn grain on an InAs NW, we demonstrate the superconducting size and hybridization of superconductivity to the semiconducting base. In brief, along with the optimal growth conditions, choosing the right lattice template is the key to realizing the desired Sn phases. Therefore, this work presents a crucial development to achieve a selective Sn phase in the hybrid and, thus, to determine the performance of NW–Sn-based topological and superconducting quantum devices.

## Methods

### Hybrid Crystal Growth

#### Molecular Beam Epitaxy

A Vecco Gen II molecular beam epitaxy device connected to a high-vacuum electron gun assisted physical vapor deposition system was used to grow NW–Sn hybrids. III–V NWs were grown in the MBE chamber; later the samples were transferred into the PVD chamber through the high-vacuum tunnel to grow Sn on the selected facets. All of the NW batches were grown on an InAs (111)B single-side polished substrate. Au was used as growth catalyst and deposited *in situ* in the MBE chamber before NW growth. In the MBE, material cells (III–V and Au) were preheated and stabilized before the growth. Optimized cell parameters for NW growth are In, 865/815 °C, As, 400/345 °C, Sb, 680/500 °C, and Au, 1100 °C. While loading the InAs (111)B substrate, standard 200 °C for 2 h of baking at a load-lock chamber followed by 250 °C for 1 h degassing at the buffer tunnel was carried out, confirming a clean InAs surface for the growth. Subsequently, the samples were transferred to the MBE growth chamber, where annealing was performed for 6 min at a 610 °C substrate temperature under high As_4_ overpressure. Next, the substrate was cooled down to 590 °C and Au was deposited for 1 s. After Au deposition, the substrates were further lowered to growth temperature, which is 447 °C, and the In flux was introduced in the growth chamber to nucleate the Au catalysts. For InAs NWs a 35 min growth was performed. For InSb and InAsSb NWs, Sb flux was introduced after the InAs stem section. Usually the stem was grown for 6 min. For InSb, the As flux was turned off after the stem, whereas for InAsSb, As flux was compensated according to the desired Sb:As ratio of the NW. The substrate was cooled to 150 °C after the NW growth and transferred to the PVD chamber for hybridizing with Sn.

#### Electron-Beam Physical Vapor Deposition

For low-temperature growth of the Sn thin film, substrate cooling was carried out with a liquid nitrogen supply. The lowest holder temperature reach of our system is approximately ∼−150 °C, which is recorded with the thermocouple connected at the back of the substrate holder. Hence, we point out that the real temperature on the sample surface may slightly deviate from the reading. Once the desired growth temperature was reached, we waited further to completely stabilize the temperature before preparing the chamber for deposition. Subsequently, the substrate was aligned with respect to the Sn source, and the deposition angle was corrected to achieve selected facets (two or three) growth. Details about NW facet selection growth and angle corrections are discussed in ref (11). Next, the source Sn materials were heated up with energetic electron beams, and the evaporated Sn flux rate was monitored through a quartz-crystal microbalance (QCM). Once our desired flux rate was achieved, Sn was deposited on the NW facets. The deposition rate was experimented from 0.5 to 5 Å/s to find the optimized morphology. After deposition, the hybrid NW sample was kept on the cold holder for a while and then unloaded through the load-lock. During the unloading process, the NW was initially exposed to oxygen for approximately 15 min and later vented with nitrogen before introducing to room temperature.

### Morphological and Structural Characterization

#### Scanning Electron Microscopy

A JEOL JSM-7800F SEM was used to investigate the morphology of the hybrid NW. Standard operating conditions for these samples are a 15 kV accelerating voltage and a 10–15 mm working distance with a secondary electron detector. Depending on the imaging requirements and investigating materials, we also used a 30° tilted viewing angle. Further, for a detailed surface morphology investigation of the hybrid facets we used a 2–5 kV accelerating voltage with a 5 mm working distance, which helped us gain information about the roughness and residual metals.

#### (Scanning) Transmission Electron Microscopy

For structural characterization with (S)TEM, NWs were directly transferred to copper grids by a simple dry transfer technique with a clean room wipe. HRTEM micrographs were acquired in a TECNAI F20 microscope operated at 200 kV. Atomically resolved HAADF data were acquired in a probe-corrected TITAN microscope operated at 300 kV, and a Wiener filter was applied to reduce noise in the displayed images. EELS mapping was carried out in a GATAN QUANTUM spectrometer coupled to a TECNAI F20 microscope operated at 200 kV with an approximately 2 eV energy resolution and 1 eV energy dispersion, collecting the range of 350–2398 eV of energy loss. Principal component analysis (PCA) was applied to the spectrum images to enhance the S/N ratio. In M_4,5_, Sn M_4,5_, Sb M_4,5_, and As L_2,3_ were the edges employed for areal density mapping. 2D and 3D atomic models were created using Rhodius software from the University of Cadiz.^60^ For the presented cross-sections, NWs were transferred to a SiO_*x*_ substrate using a micromanipulator, where a 100 nm tungsten needle was used to selectively pick the NWs and place them parallel to each other. Next, an atomic layer deposition system was used to deposit 30 nm of AlO_*x*_ as a protective layer. A focused ion beam (Helios 600 dual beam microscope) was used to prepare cross-sectional NW sections.

### Device Fabrication

NW–Sn devices were fabricated on a highly p-doped Si/SiO_2_ substrate with a predefined alignment grid. Individual NWs were transferred from the growth wafer to the device substrate with a mechanical micromanipulator carrying a 100 nm tungsten needle and imaged with SEM to define their exact location. Contacts were patterned using electron-beam lithography and e-beam evaporation of Ti/Au (5 nm/300 nm). A6 resist was used for spin coating for 45 s with 4000 rpm and pumped in a vacuum chamber for 3 h. Patterns were developed in 1:3 MIBK/IPA for 45 s, passivated in IPA for 30 s, and washed with oxygen plasma for 30 s. Prior metal evaporation RF Ar ion milling was employed with 7 W for 5 min at an 18 mTorr pressure. The deposition rate of Ti and Au was 0.1 and 0.3 nm/s, respectively. Acetone was used for lift-off. For the devices of Figure 5(b), the distance of the inner probes was defined by the design software at 250–500 nm.

## Supporting Information Available

S1. Unit cells and crystal structure parameters of InSb, InAsSb, InAs, α-Sn, and β-Sn; S2. InSb–Sn morphology and EELS map; S3. FIB- or ALD-induced damage during cross-section preparation; S4. InSb–Sn: β-Sn grain formation within α-Sn shell; S5. Temperature effect on the hybrid structure; S6. InSb–Sn grown at room temperature; S7. InSb–Sn grown at sample holder temperature −50 °C; S8. InSb–Sn grown at sample holder temperature −100 °C; S9. InSb–Sn grown at sample holder temperature −150 °C; S10. Additional HRTEM micrographs of 3 nm Sn on InSb NW; S11. Defect formation and initial β-Sn nucleation in a 5 nm Sn shell on an InSb NW; S12. Additional micrographs of a 40 nm Sn shell on an InSb NW; S13. Thermal stability of the α-β-Sn/InSb heterostructure; S14. Additional SEM images of InAsSb–Sn morphology; S15. Additional structural analysis of InAsSb/Sn; S16. Structural analysis of InAs/Sn NWs; S17. β-Sn phase analysis on an InAs NW; S18. Grain boundary within β-Sn on InAs facets; S19. Additional β-Sn analysis on am InAs NW; S20. Additional β-Sn orientations on an InAs NW; S21. InSb–InAs(2 nm)–Sn; S22; Summary of 3D models of the α-β/InX configuration; S23. Growth parameters of the fabricated devices; S24. Additional device measurement (PDF)

## References

[ref1] Dean R.; Thwaites C. Tinplate and Tin Coating Technology. JOM 1987, 39, 42–45. 10.1007/BF03258609.

[ref2] Ng M.-F.; Tan T. L. Unveiling stable group IV alloy nanowires via a comprehensive search and their electronic band characteristics. Nano Lett. 2013, 13, 4951–4956. 10.1021/nl402987c. 23984910

[ref3] Küfner S.; Furthmüller J.; Matthes L.; Fitzner M.; Bechstedt F. Structural and electronic properties of α-tin nanocrystals from first principles. Phys. Rev. B 2013, 87, 235307 10.1103/PhysRevB.87.235307.

[ref4] Küfner S.; Furthmüller J.; Matthes L.; Bechstedt F. Optical absorption and emission of α-Sn nanocrystals from first principles. Nanotechnology 2013, 24, 405702 10.1088/0957-4484/24/40/405702. 24029081

[ref5] Black H. Getting the lead out of electronics. Environmental Health Sciences 2005, 113, 682–685. 10.1289/ehp.113-a682. PMC1281311 16203230

[ref6] Hörmann N. G.; Gross A.; Kaghazchi P. Semiconductor–metal transition induced by nanoscale stabilization. Phys. Chem. Chem. Phys. 2015, 17, 5569–5573. 10.1039/C4CP05619A. 25626452

[ref7] Tu Z.; Choudhury S.; Zachman M. J.; Wei S.; Zhang K.; Kourkoutis L. F.; Archer L. A. Fast ion transport at solid–solid interfaces in hybrid battery anodes. Nature Energy 2018, 3, 310–316. 10.1038/s41560-018-0096-1.

[ref8] Xu C.-Z.; Chan Y.-H.; Chen Y.; Chen P.; Wang X.; Dejoie C.; Wong M.-H.; Hlevyack J. A.; Ryu H.; Kee H.-Y.; et al. Elemental topological Dirac semimetal: α-Sn on InSb (111). Physical review letters 2017, 118, 146402 10.1103/PhysRevLett.118.146402. 28430465

[ref9] Ohtsubo Y.; Le Fevre P.; Bertran F.; Taleb-Ibrahimi A. Dirac cone with helical spin polarization in ultrathin α-Sn (001) films. Physical review letters 2013, 111, 216401 10.1103/PhysRevLett.111.216401. 24313507

[ref10] Pendharkar M.; Zhang B.; Wu H.; Zarassi A.; Zhang P.; Dempsey C.; Lee J.; Harrington S.; Badawy G.; Gazibegovic S.; et al. Parity-preserving and magnetic field–resilient superconductivity in InSb nanowires with Sn shells. Science 2021, 372, 508–511. 10.1126/science.aba5211. 33858990

[ref11] Khan S. A.; Lampadaris C.; Cui A.; Stampfer L.; Liu Y.; Pauka S. J.; Cachaza M. E.; Fiordaliso E. M.; Kang J.-H.; Korneychuk S.; et al. Highly transparent gatable superconducting shadow junctions. ACS Nano 2020, 14, 14605–14615. 10.1021/acsnano.0c02979. 32396328

[ref12] Busch G.; Kebn R. Semiconducting Properties of Gray Tin; Solid State Physics; Academic Press: New York, 1960; Vol. 11; pp 1–40.

[ref13] Ewald A.; Tufte O. Gray tin single crystals. J. Appl. Phys. 1958, 29, 1007–1009. 10.1063/1.1723351.

[ref14] Groves S.; Paul W. Band structure of gray tin. Phys. Rev. Lett. 1963, 11, 194 10.1103/PhysRevLett.11.194.

[ref15] Farrow R.; Robertson D.; Williams G.; Cullis A.; Jones G.; Young I.; Dennis P. The growth of metastable, heteroepitaxial films of α-Sn by metal beam epitaxy. J. Cryst. Growth 1981, 54, 507–518. 10.1016/0022-0248(81)90506-6.

[ref16] Legrain F.; Manzhos S. Understanding the difference in cohesive energies between alpha and beta tin in DFT calculations. AIP Advances 2016, 6, 045116 10.1063/1.4948434.

[ref17] Didschuns I.; Fleischer K.; Schilbe P.; Esser N.; Richter W.; Lüders K. Superconductivity in Sn films on InSb (110) taking account of the film morphology and structure. Physica C: Superconductivity 2002, 377, 89–95. 10.1016/S0921-4534(01)01121-2.

[ref18] Molodets A. M.; Nabatov S. S. Thermodynamic potentials, diagram of state, and phase transitions of tin on shock compression. High Temperature 2000, 38, 715–721. 10.1007/BF02755923.

[ref19] Smith R. W. The α(semiconductor) ag β(metal) transition in tin. Journal of the Less Common Metals 1985, 114, 69–80. 10.1016/0022-5088(85)90391-1.

[ref20] De Haas W.; De Boer J.; Van den Berg G. The electrical resistance of cadmium, thallium and tin at low temperatures. Physica 1935, 2, 453–459. 10.1016/S0031-8914(35)90114-8.

[ref21] Song H.; Yao J.; Ding Y.; Gu Y.; Deng Y.; Lu M.-H.; Lu H.; Chen Y.-F. Thermal stability enhancement in epitaxial alpha tin films by strain engineering. Adv. Eng. Mater. 2019, 21, 1900410 10.1002/adem.201900410.

[ref22] Roman B. J.; Ewald A. Stress-induced band gap and related phenomena in gray tin. Phys. Rev. B 1972, 5, 3914 10.1103/PhysRevB.5.3914.

[ref23] Osaka T.; Omi H.; Yamamoto K.; Ohtake A. Surface phase transition and interface interaction in the α-Sn/InSb 111 system. Phys. Rev. B 1994, 50, 7567 10.1103/PhysRevB.50.7567. 9974738

[ref24] Fu L.; Kane C. L. Topological insulators with inversion symmetry. Phys. Rev. B 2007, 76, 045302 10.1103/PhysRevB.76.045302.

[ref25] Barfuss A.; Dudy L.; Scholz M. R.; Roth H.; Höpfner P.; Blumenstein C.; Landolt G.; Dil J.; Plumb N.; Radovic M.; et al. Elemental topological insulator with tunable Fermi level: Strained α-Sn on InSb (001). Physical review letters 2013, 111, 157205 10.1103/PhysRevLett.111.157205. 24160626

[ref26] Zheng X.; Zhang J.-F.; Tong B.; Du R.-R. Epitaxial growth and electronic properties of few-layer stanene on InSb (1 1 1). 2D Materials 2020, 7, 011001 10.1088/2053-1583/ab42b9.

[ref27] Majorana E. Teoria simmetrica dell’elettrone e del positrone. Nuovo Cimento 1937, 5, 171–184. 10.1007/BF02961314.

[ref28] Kitaev A. Y. Unpaired Majorana fermions in quantum wires. Physics-Uspekhi 2001, 44, 131–136. 10.1070/1063-7869/44/10S/S29.

[ref29] Lutchyn R. M.; Sau J. D.; Sarma S. D. Majorana fermions and a topological phase transition in semiconductor-superconductor heterostructures. Phys. Rev. Lett. 2010, 105, 077001 10.1103/PhysRevLett.105.077001. 20868069

[ref30] Oreg Y.; Refael G.; von Oppen F. Helical liquids and Majorana bound states in quantum wires. Phys. Rev. Lett. 2010, 105, 177002 10.1103/PhysRevLett.105.177002. 21231073

[ref31] Mourik V.; Zuo K.; Frolov S. M.; Plissard S. R.; Bakkers E. P. A. M.; Kouwenhoven L. P. Signatures of Majorana Fermions in Hybrid Superconductor-Semiconductor Nanowire Devices. Science 2012, 336, 1003–1007. 10.1126/science.1222360. 22499805

[ref32] Rokhinson L. P.; Liu X.; Furdyna J. K. The fractional a.c. Josephson effect in a semiconductor–superconductor nanowire as a signature of Majorana particles. Nat. Phys. 2012, 8, 795–799. 10.1038/nphys2429.

[ref33] Deng M. T.; Vaitiekenas S.; Hansen E. B.; Danon J.; Leijnse M.; Flensberg K.; Nygard J.; Krogstrup P.; Marcus C. M. Majorana bound state in a coupled quantum-dot hybrid-nanowire system. Science 2016, 354, 1557–1562. 10.1126/science.aaf3961. 28008065

[ref34] Das A.; Ronen Y.; Most Y.; Oreg Y.; Heiblum M.; Shtrikman H. Zero-bias peaks and splitting in an Al–InAs nanowire topological superconductor as a signature of Majorana fermions. Nat. Phys. 2012, 8, 887–895. 10.1038/nphys2479.

[ref35] Lutchyn R. M.; Bakkers E. P. A. M.; Kouwenhoven L. P.; Krogstrup P.; Marcus C. M.; Oreg Y. Majorana zero modes in superconductor–semiconductor heterostructures. Nature Reviews Materials 2018, 3, 52–68. 10.1038/s41578-018-0003-1.

[ref36] Frolov S.; Manfra M.; Sau J. Topological superconductivity in hybrid devices. Nat. Phys. 2020, 16, 718–724. 10.1038/s41567-020-0925-6.

[ref37] Leijnse M.; Flensberg K. Introduction to topological superconductivity and Majorana fermions. Semicond. Sci. Technol. 2012, 27, 124003 10.1088/0268-1242/27/12/124003.

[ref38] Prada E.; San-Jose P.; de Moor M. W.; Geresdi A.; Lee E. J.; Klinovaja J.; Loss D.; Nygård J.; Aguado R.; Kouwenhoven L. P. From Andreev to Majorana bound states in hybrid superconductor–semiconductor nanowires. Nature Reviews Physics 2020, 2, 1–20. 10.1038/s42254-020-0228-y.

[ref39] Liu S.; Covian A. C.; Gardener J. A.; Akey A.; Levin B. D.; Wang X.; Liu J. Growth of α-Sn on silicon by a reversed β-Sn to α-Sn phase transformation for quantum material integration. Communications Materials 2022, 3, 1–11. 10.1038/s43246-022-00241-7.

[ref40] Höchst H.; Calderón I. H. Microscopic electronic structure and growth mode of Sn/InSb (111) interfaces. Journal of Vacuum Science & Technology A: Vacuum, Surfaces, and Films 1985, 3, 911–914. 10.1116/1.573347.

[ref41] Oehl N.; Hardenberg L.; Knipper M.; Kolny-Olesiak J.; Parisi J.; Plaggenborg T. Critical size for the β-to α-transformation in tin nanoparticles after lithium insertion and extraction. CrystEngComm 2015, 17, 3695–3700. 10.1039/C5CE00148J.

[ref42] Burgers W.; Groen L. Mechanism and kinetics of the allotropic transformation of tin. Discuss. Faraday Soc. 1957, 23, 183–195. 10.1039/df9572300183.

[ref43] Musgrave M. On the relation between grey and white tin (α -Sn and β-Sn). Proc. R. Soc. London. Ser. A 1963, 272, 503–528.

[ref44] Ravelo R.; Baskes M. Equilibrium and thermodynamic properties of grey, white, and liquid tin. Phys. Rev. Lett. 1997, 79, 2482 10.1103/PhysRevLett.79.2482.

[ref45] Yomogita K. Geometry of the Crystal Lattice of β-Tin. Jpn. J. Appl. Phys. 1972, 11, 1 10.1143/JJAP.11.1.

[ref46] Ojima K.; Taneda Y.; Takasaki A. Direct observation of α → βtransformation in tin by transmission electron microscopy. physica status solidi (a) 1993, 139, 139–144. 10.1002/pssa.2211390111.

[ref47] de la Mata M.; Magen C.; Gazquez J.; Utama M. I. B.; Heiss M.; Lopatin S.; Furtmayr F.; Fernández-Rojas C. J.; Peng B.; Morante J. R.; Rurali R.; Eickhoff M.; Fontcuberta i Morral A.; Xiong Q.; Arbiol J. Polarity Assignment in ZnTe, GaAs, ZnO, and GaN-AlN Nanowires from Direct Dumbbell Analysis. Nano Lett. 2012, 12, 2579–2586. 10.1021/nl300840q. 22493937

[ref48] de la Mata M.; Zamani R. R.; Martí-Sánchez S.; Eickhoff M.; Xiong Q.; Fontcuberta i Morral A.; Caroff P.; Arbiol J. The Role of Polarity in Nonplanar Semiconductor Nanostructures. Nano Lett. 2019, 19, 3396–3408. 10.1021/acs.nanolett.9b00459. 31039314

[ref49] Khan S. A.; Stampfer L.; Mutas T.; Kang J.-H.; Krogstrup P.; Jespersen T. S. Multiterminal Quantized Conductance in InSb Nanocrosses. Adv. Mater. 2021, 33, 2100078 10.1002/adma.202100078. 34075631

[ref50] Carrad D. J.; Stampfer L.; Olsteins D.; Petersen C. E. N.; Khan S. A.; Krogstrup P.; Jespersen T. S. Photon-Assisted Tunneling of High-Order Multiple Andreev Reflections in Epitaxial Nanowire Josephson Junctions. Nano Lett. 2022, 22, 6262–6267. 10.1021/acs.nanolett.2c01840. 35862144

[ref51] Stampfer L.; Carrad D. J.; Olsteins D.; Petersen C. E.; Khan S. A.; Krogstrup P.; Jespersen T. S. Andreev Interference in the Surface Accumulation Layer of Half-Shell InAsSb/Al Hybrid Nanowires. Adv. Mater. 2022, 34, 2108878 10.1002/adma.202108878. 35050545

[ref52] Thompson C.; Floro J.; Smith H. I. Epitaxial grain growth in thin metal films. Journal of applied physics 1990, 67, 4099–4104. 10.1063/1.344969.

[ref53] Krogstrup P.; Ziino N.; Chang W.; Albrecht S.; Madsen M.; Johnson E.; Nygård J.; Marcus C.; Jespersen T. Epitaxy of semiconductor–superconductor nanowires. Nat. Mater. 2015, 14, 400–406. 10.1038/nmat4176. 25581626

[ref54] Vegard L. Die konstitution der mischkristalle und die raumfüllung der atome. Zeitschrift für Physik 1921, 5, 17–26. 10.1007/BF01349680.

[ref55] Martí-Sánchez S.; Botifoll M.; Oksenberg E.; Koch C.; Borja C.; Spadaro M. C.; Di Giulio V.; Ramasse Q.; García de Abajo F. J.; Joselevich E.; et al. Sub-nanometer mapping of strain-induced band structure variations in planar nanowire core-shell heterostructures. Nat. Commun. 2022, 13, 1–10. 10.1038/s41467-022-31778-3. 35835772 PMC9283334

[ref56] Spirkoska D.; Arbiol J.; Gustafsson A.; Conesa-Boj S.; Glas F.; Zardo I.; Heigoldt M.; Gass M. H.; Bleloch A. L.; Estrade S.; Kaniber M.; Rossler J.; Peiro F.; Morante J. R.; Abstreiter G.; Samuelson L.; Fontcuberta i Morral A. Structural and optical properties of high quality zinc-blende/wurtzite GaAs nanowire heterostructures. Phys. Rev. B 2009, 80, 245325 10.1103/PhysRevB.80.245325.

[ref57] Arbiol J.; Fontcuberta i Morral A.; Estradé S.; Peiró F.; Kalache B.; Roca i Cabarrocas P.; Morante J. R. Influence of the (111) twinning on the formation of diamond cubic/diamond hexagonal heterostructures in Cu-catalyzed Si nanowires. J. Appl. Phys. 2008, 104, 064312 10.1063/1.2976338.

[ref58] Lang X.; Jiang Q. Finite size effect on critical transition temperature of superconductive nanosolids. Solid state communications 2005, 134, 797–801. 10.1016/j.ssc.2005.03.039.

[ref59] Han Z.; Allain A.; Arjmandi-Tash H.; Tikhonov K.; Feigel’man M.; Sacépé B.; Bouchiat V. Collapse of Superconductivity in a Hybrid Tin–Graphene Josephson Junction Array. Nature Phys. 2014, 10, 380–386. 10.1038/nphys2929.

[ref60] Bernal S.; Botana F.; Calvino J.; López-Cartes C.; Pérez-Omil J.; Rodriguez-Izquierdo J. The interpretation of HREM images of supported metal catalysts using image simulation: profile view images. Ultramicroscopy 1998, 72, 135–164. 10.1016/S0304-3991(98)00009-6.

