# Supplementary material for: Epitaxially Driven Phase Selectivity of Sn in Hybrid Quantum Nanowires
Source: ACS Nano. 2023 Jun 15;17(12):11794–804. doi: 10.1021/acsnano.3c02733 (PMC10311604; doi:10.1021/acsnano.3c02733)
Supplement: Supplementary file 1 — nn3c02733_si_001.pdf [file nn3c02733_si_001.pdf]

Supporting Information

Epitaxially Driven Phase Selectivity of Sn in Hybrid Quantum Nanowires

Sabbir A. Khan,[*,†,‡,#] Sara Martí-Sánchez,[*,¶,#] Dags Olsteins,[†] Charalampos Lampadaris,[†] Damon James Carrad,[†,§] Yu Liu,[†] Judith Quiñones,[¶] Maria Chiara Spadaro,[¶] Thomas Sand Jespersen,[†,§] Peter Krogstrup,[*,∥] and Jordi Arbiol[*,¶,⊥]

†*Center for Quantum Devices, Niels Bohr Institute, University of Copenhagen, 2100 Copenhagen, Denmark*

‡*Danish Fundamental Metrology, Kogle Alle 5, 2970 Hørsholm, Denmark*

¶*Catalan Institute of Nanoscience and Nanotechnology (ICN2), CSIC and BIST, Campus UAB, Bellaterra, 08193 Barcelona, Catalonia, Spain*

§*Department of Energy Conversion and Storage, Technical University of Denmark, Fysikvej, Building, Lyngby, 310, 2800 Denmark*

∥*NNF Quantum Computing Programme, Niels Bohr Institute, University of Copenhagen, 2100 Copenhagen, Denmark*

⊥*ICREA, Pg. Lluís Companys 23, 08010 Barcelona, Catalonia, Spain*

#*Contributed equally to this work*

E-mail: skh@dfm.dk; sara.marti@icn2.cat; krogstrup@nbi.dk; arbiol@icrea.cat

S1. Unit cells and crystal structure parameters of InSb, InAsSb, InAs, α-Sn and β-Sn

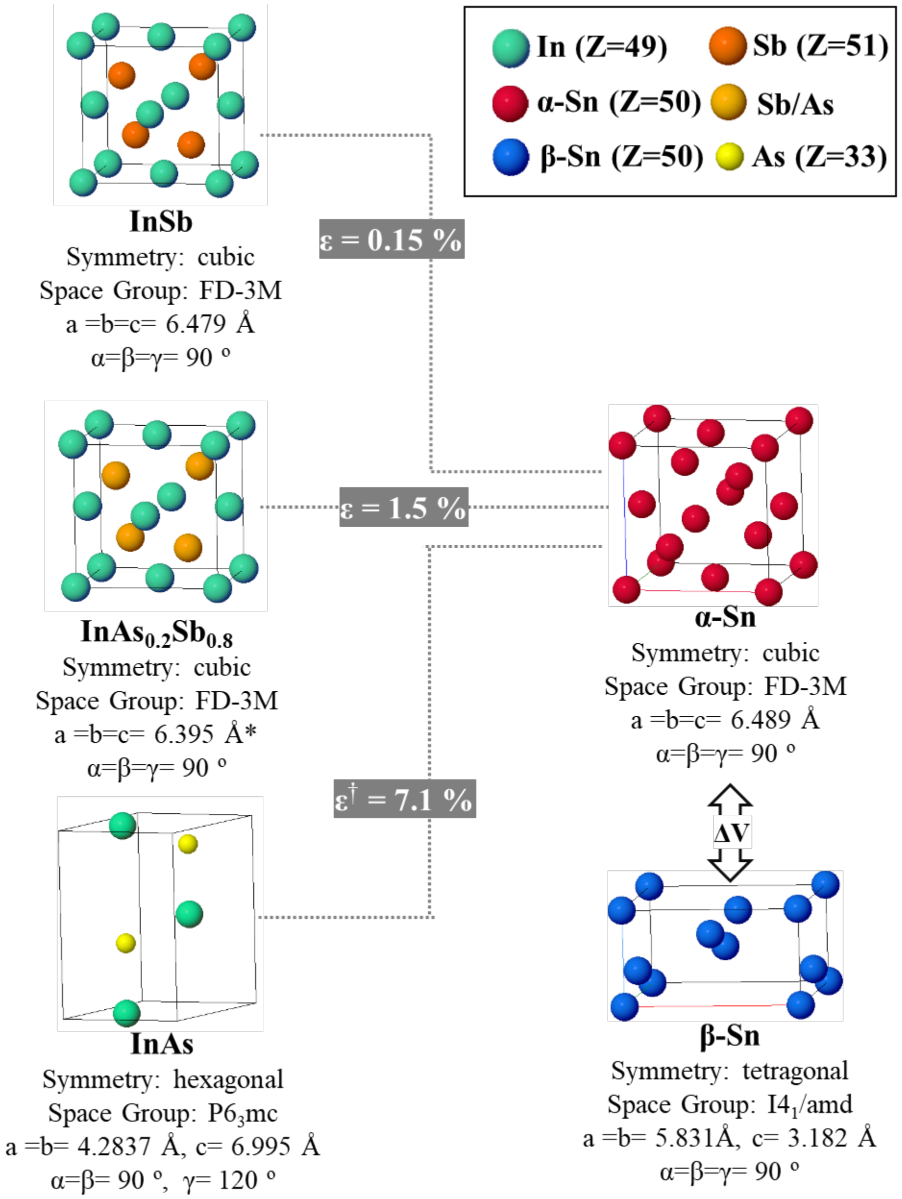

Figure S1: Unit cell and crystal structure parameters of InSb, InAsSb* , InAs, α-Sn[1] and β-Sn.[2] The associated lattice mismatch between the NW materials (InSb, InAsSb and InAs†) and α-Sn are specified.

*Lattice constant calculated from Vegard's law[3]

†Mismatch calculated between InAs(0002) planes and α-Sn (111) planes, given the equivalences between < 111 > cubic and < 0001 > hexagonal configurations.[4,5]

S2. InSb-Sn morphology and EELS map

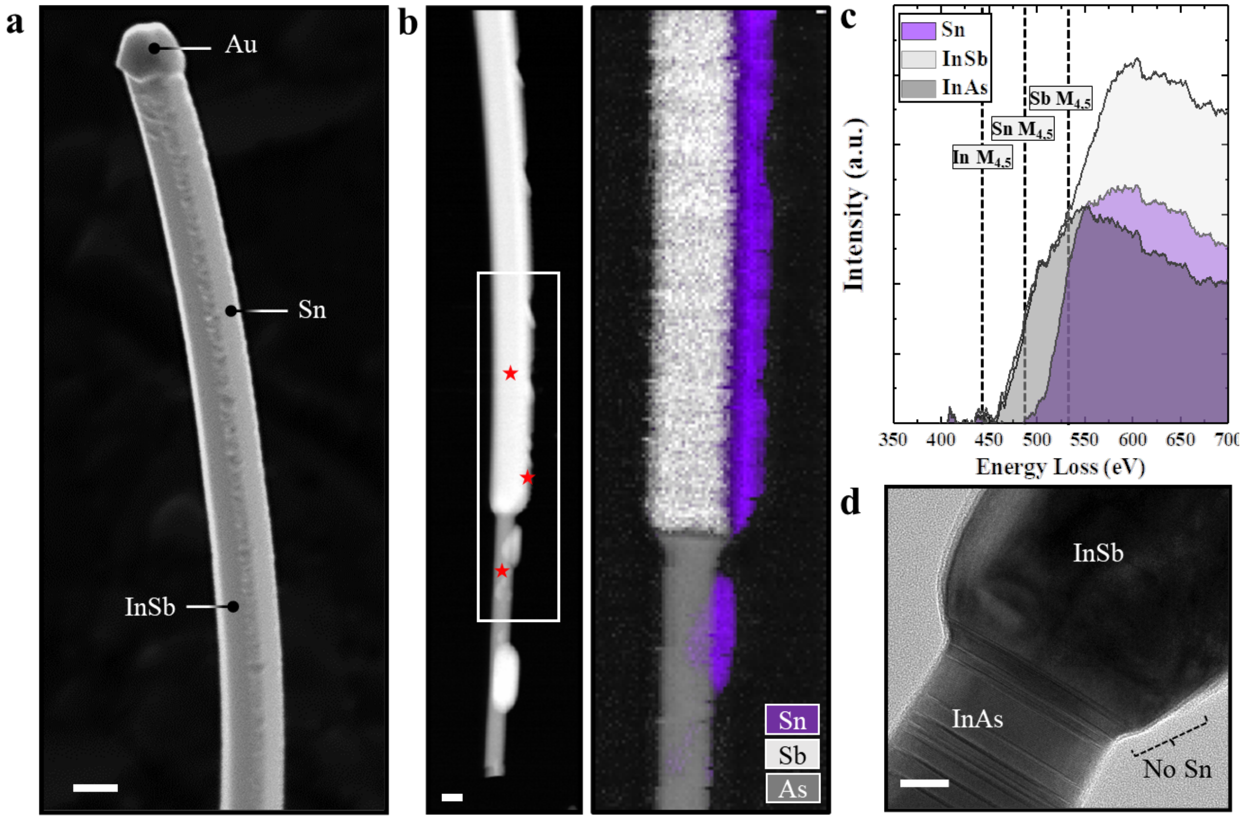

Figure S2: **a**, SEM image of the long InSb-Sn section. A small bending of the NW away from Sn deposition direction is observed. This can be attributed to either different thermal expansion coefficient of both materials or residual strain in the interface.[6,7] **b**, EELS elemental map of Sn, Sb and As on the InSb/InAs section of the NWs. A continuous Sn shell can be appreciated on the InSb surface, while it deposits as discrete islands on the InAs stem. **c**, EELS spectra extracted from the regions marked in (b) after background subtraction with In, Sn and Sb edges marked. **d**, Bottom curvature in the intersection area between InAs and InSb . Sn is typically absent in this area. This is possibly due to the high surface energy in the curved area or the region might be shadowed when Sn is deposited given the curvature. Scale bars are: (a) 100 nm, (b) 100 nm, (d) 20 nm.

S3. FIB or ALD-induced damage during cross-section preparation

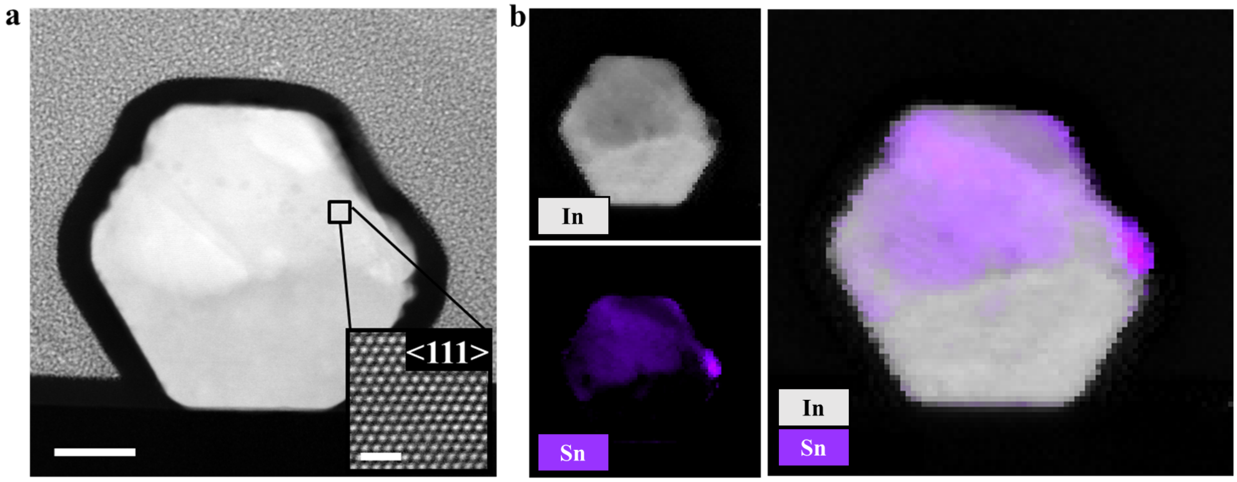

Figure S3: **a**, HAADF-STEM overview of a FIB cross section presenting anomalous contrast and a deformation of the hexagonal cross-section morphology. Nevertheless, the atomic arrangement is unaltered and matches that of <111> oriented cubic ZB/diamond crystal structure. **b**, EELS-based elemental mapping of the same area of the HAADF micrograph of In and Sn. Higher Sn concentration is observed at the top-right facet of the NW, where it was originally deposited, but it migrates to the inside of the NW. This migration is possibly happening due to the high temperature exposure during ALD process or during FIB preparation. Hence, during the Sn based NW device preparation, careful considerations are required on the thermal budget of the different process steps. Scale bars are: 50 nm (main), 1 nm (inset).

S4. InSb-Sn: β-Sn grain formation within α-Sn shell

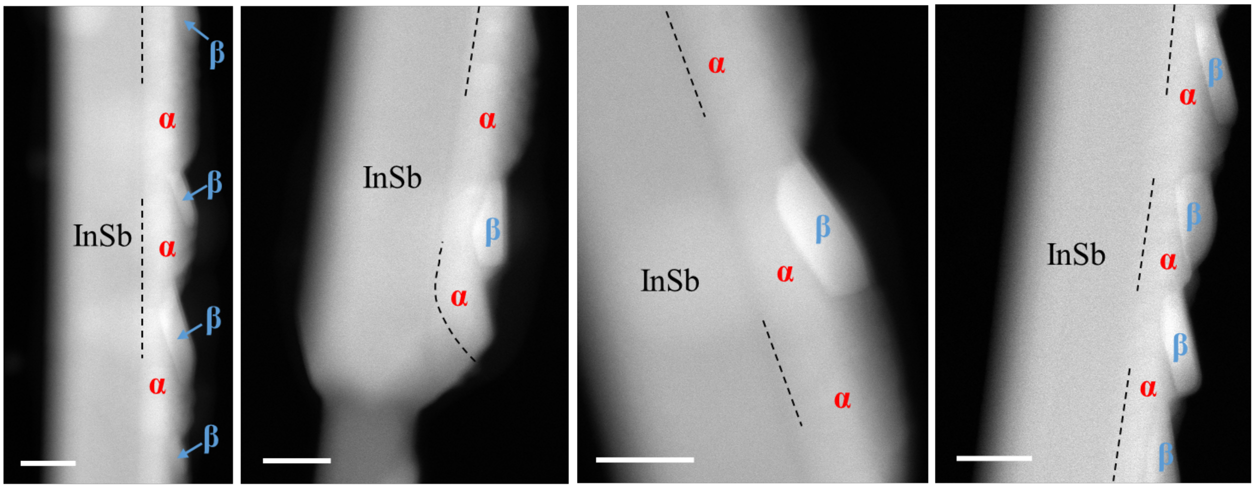

Figure S4: Low magnification HAADF-STEM micrographs of InSb - Sn (20 nm) nanowires grown at -100 °C. The positioning of the $\alpha-$Sn shell on the InSb core can be observed by an increase in HAADF intensity (brighter). Dashed lines are added as eye guides to show these positions. $\alpha-$Sn grows in direct contact with the InSb nanowire core and $\beta-$Sn grains can be observed to grow embedded in $\alpha-$Sn. Scale bars are 50 nm.

S5. Temperature effect on the hybrid structure

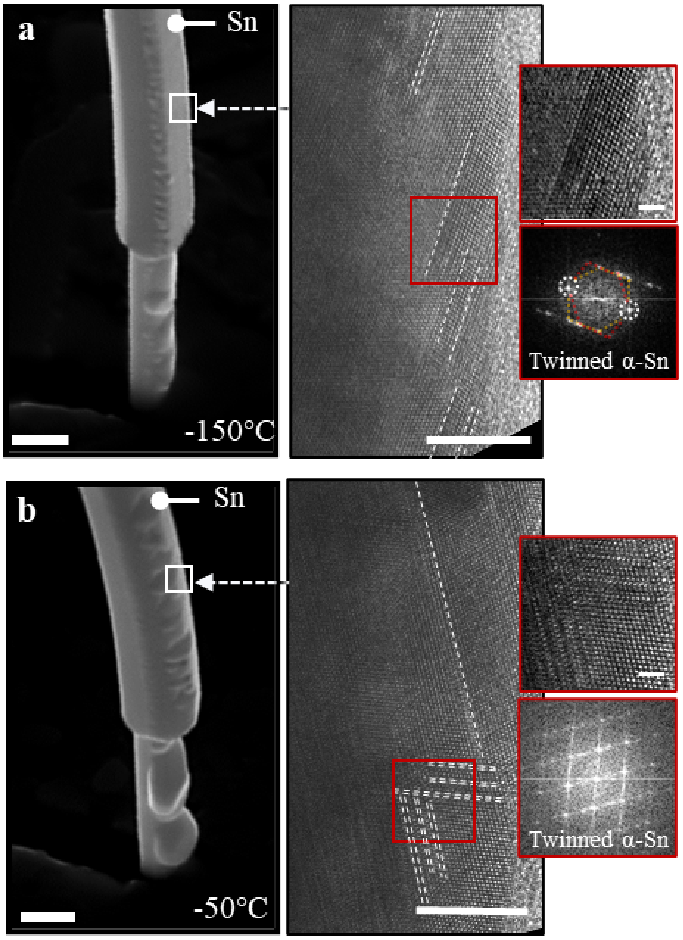

Figure S5: **a**, Tilted SEM image of the hybrid InSb/Sn grown at substrate temperature approximately -150°C. To the right HRTEM analysis of the α-Sn structure. Here planar defects (twins or stacking faults) are mostly observed in the {111} planes oblique to the growth direction. **b**, Tilted SEM image of the hybrid heterostructure where substrate temperature is approximately -50°C. Discrete Sn morphology is observed growing at this temperature. HRTEM analysis of the $\alpha - Sn$ structure reveals defects in all available {111} planes unlike panel (a). Scale bars in SEM (a), (b): 100 nm and HRTEM: 10 nm, 2 nm.

Fig.S5 shows the evolution of Sn film on InAs stem-assisted InSb NWs at different substrate temperatures. Stem-assisted multi-stacking growth helps us to evaluate Sn shells on both materials. Sn growth temperatures were varied from room temperature to -150 °C, and 20 nm of Sn were deposited on the NWs. Fig. S5 shows the structural arrangement of $\alpha -$ Sn phase for Sn grown at -50°C and -150°C. In addition, room temperature and -100°C

growth batch with corresponding morphology are presented in the S6 and S8, with additional HAADF micrographs of -100°C NWs at S4. At -50° C substrate temperature, deposited Sn on InSb NW facets exhibits discrete and rough morphology. Here, the temperature is not low enough to confine adatom diffusion length. As a result, the adatom incorporation rate in the neighboring nucleation sites is reduced, which leads to a discrete film in the given thickness. On the other hand, the samples grown at -100°C (in S4 and S8) and -150°C (in Fig. S5 (a) and S9) exhibit a continuous Sn thin film with significantly improved uniformity on the InSb segment. At these temperatures, adatom diffusion length is substantially reduced since adatoms do not have enough kinetic energy to be mobile on the NW surface. Consequently, the incorporation rate in the adjacent nucleation sites increases and a continuous thin film forms within the initial stage, where surface-driven growth scheme dominates.[6–8] Nevertheless, compared to the -100°C grown sample, the smoothness of the Sn shell morphology further improves in -150°C sample. Please note that with the same temperature, the surface roughness will change for thicker Sn films (approx. >100 nm). After a critical thickness, the surface formation and orientation of the film will be dominated by the grain boundary and mobility, which will determine the morphology.[6–8]

In addition to the surface morphology, there is a notable structural difference observed at different temperatures. At low temperature (-150°C and -100°C) there are several twinning events in the region purely corresponding to the shell materials. These twinning events belong to the {111} plane oblique to the growth direction as shown in Fig.S5(a). Planar defects perpendicular to the growth direction are not commonly observed for low temperature samples. In contrary, comparatively high density of defects perpendicular to the growth direction and other newly formed {111} planes are observed in high temperature (-50°C and close to RT) grown Sn shell (Fig. S5(b), S6 and S7). Further, twining length seems to be much longer for high temperature Sn shells compared to the low temperature ones. Moreover, investigating several NWs from each temperature batch, we observed higher density of $\beta - \mathrm{Sn}$ grains in low temperature grown Sn shell compared to the high temperature ones.

In contrast, as shown in Fig. S5, Sn on InAs stem deposits as discrete islands and remain unaffected over different temperature ranges. As elaborated in the main-text, the interface energy of two materials is the key here. Low residual mismatch between InSb and $\alpha - \text{Sn}$ leads to minimum interface energy.[9–11] Hence, as manifested in the growth, substrate holder temperatures ≤-100°C assists Sn to wet on the InSb NW facets making an uniform thin film even in the low thickness range (approx. 3 nm). On the other hand, with large mismatch leading to high interface energy, Sn on InAs stem shows dewetted islands even at the lowest holder temperature possible (-150°C) in our MBE system. To be able to create a continuous film of Sn on InAs NW, temperature requires to be lowered further so that adatoms are kinetically locked and hence, stick on their landing site (not within the capability of this experiment). Another way is increasing the thickness. After overcoming a critical thickness, islands will impinge onto each other accumulating as a continuous film on NW facets.

S6. InSb-Sn grown at room temperature

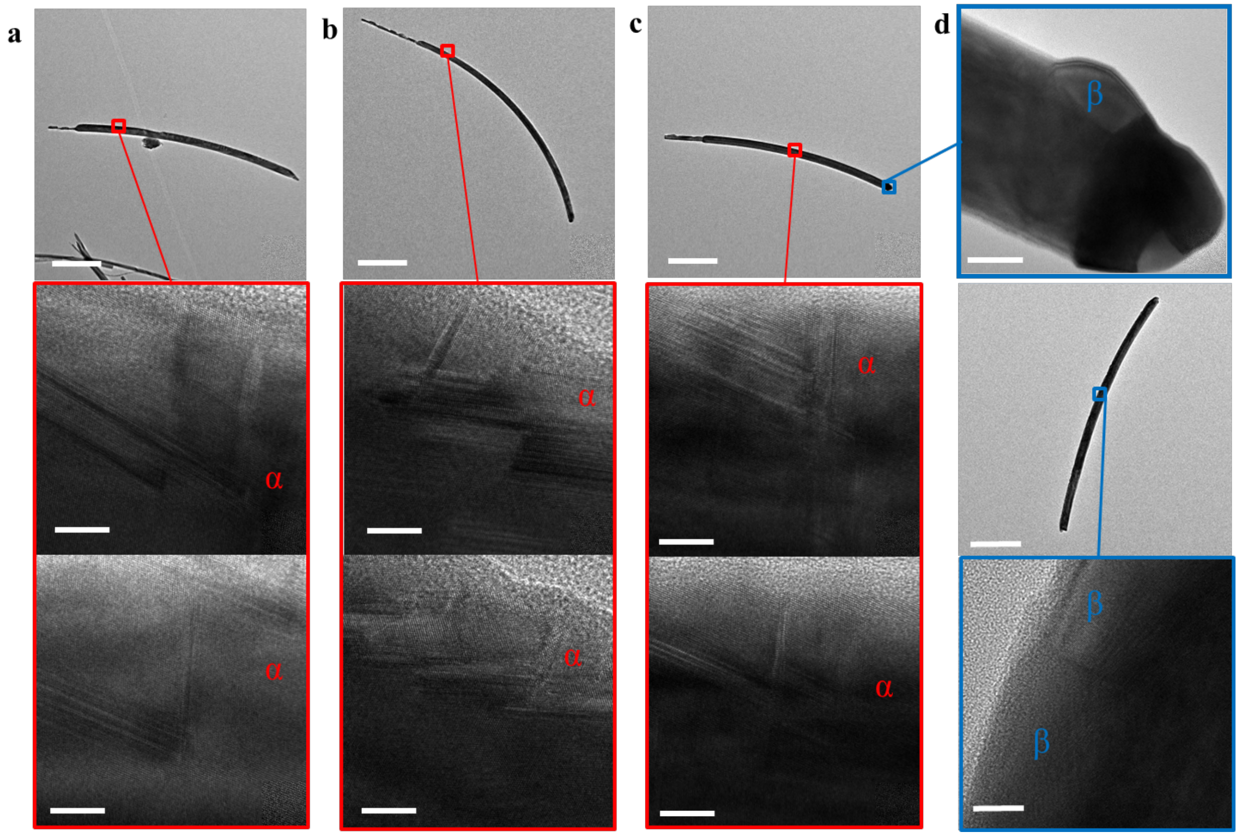

Figure S6: **a-c**, Details of majority observation of defective α - Sn in three representative NWs. Multiple planar defects in the available {111} α -Sn planes (mainly stacking faults, but also twinning) can be observed. There is frequent appearence of defects perpendicular to the growth direction. **d**, Details on the found β-Sn grains on the NWs grown at this temperature. We could only detect three different grains in the NW body, found in three different nanowires out of 10 NW examined. However, it is frequent to find β-Sn deposition in contact with the solidified Au-catalyst as the only β grain present in the whole NW heterostructure. We presume high residual mismatch between α-Sn and catalyst particle is the reason to accelerate β-Sn nucleation at Au-Sn interface. Scale bars: (a-c) top: 1 μm, middle: 10 nm, bottom: 10 nm; (d) top: 50 nm, middle: 1 μm, bottom: 10 nm.

S7. InSb-Sn grown at sample holder temperature -50°C

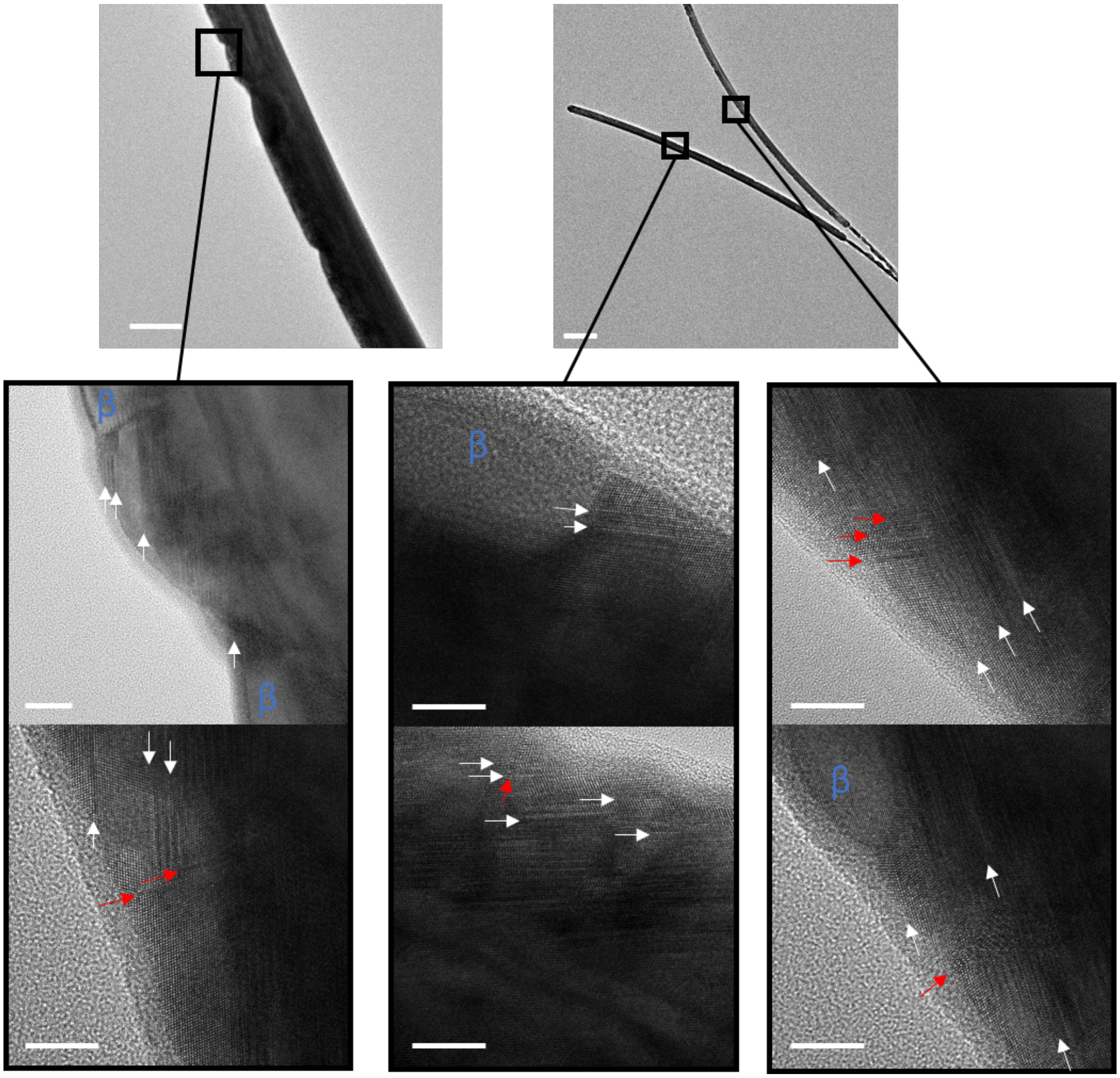

Figure S7: Additional HRTEM micrographs with focus on α-Sn phase in InSb-Sn NWs grown at -50 °C. A dominance of planar defects oblique to the NW axis is also observed. Some of these and their direction are indicated with white arrows. Additional planar defects perpendicular to the NW axis are also observed in most of the NWs (indicated with red arrows). Scale bars are: top: 50 nm & 500 nm, middle & bottom: 10 nm.

S8. InSb-Sn grown at sample holder temperature -100°C

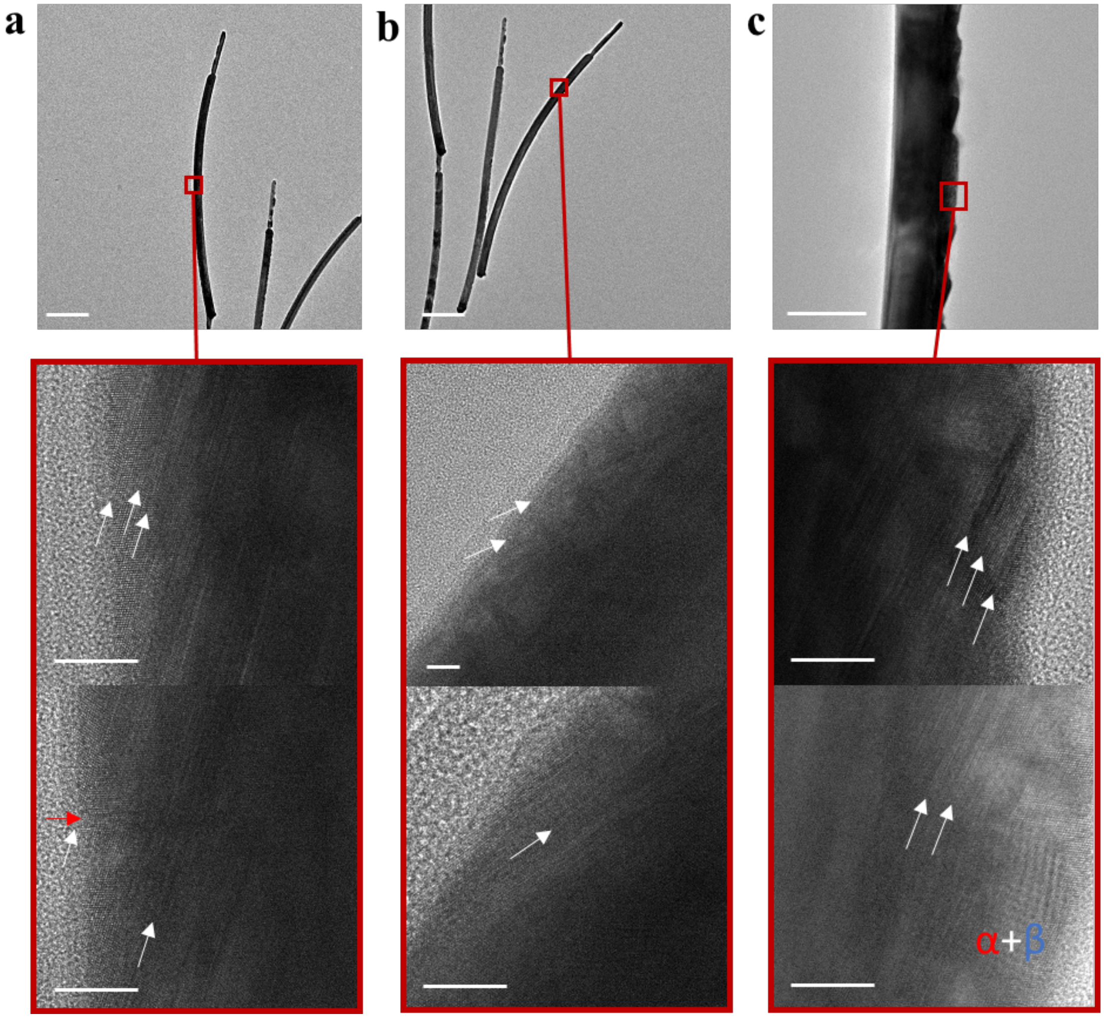

Figure S8: Additional HRTEM micrographs with focus on α-Sn phase in InSb-Sn NWs grown at -100°C. A dominance of planar defects oblique to the NW axis is observed. These dominant twin directions are indicated with white arrows. Some stacking faults perpendicular to the NW axis are present but rarely observed, as indicated with a red arrow in (a), and its average length relatively short. Scale bars are: top: 500 nm, 500 nm & 200 nm , middle & bottom: 10 nm.

S9. InSb-Sn grown at sample holder temperature -150°C

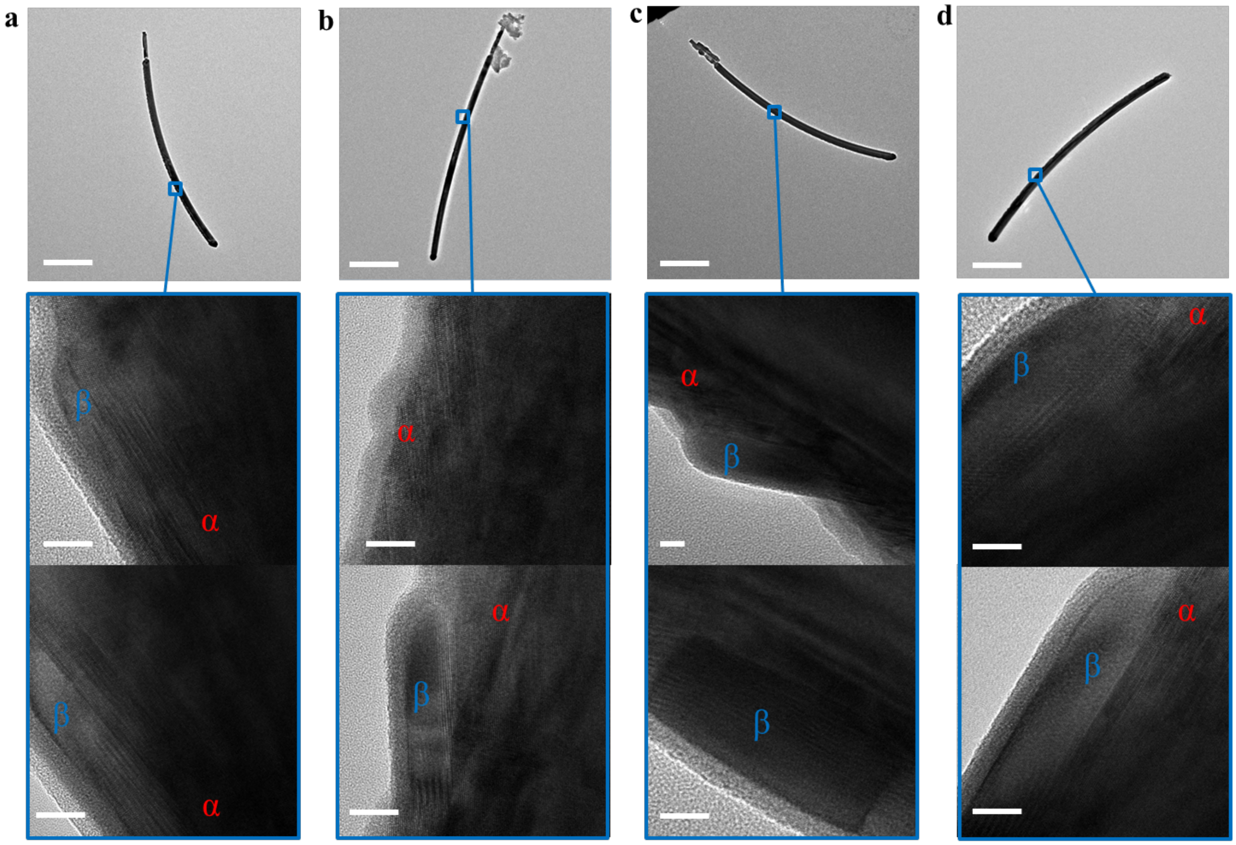

Figure S9: **a-d**, Top: overview of several NWs grown at -150°C. Bottom: details on atomic arrangement. Growth at -150°C seems to favor β-Sn nucleation, as β-Sn grains could be clearly identified in 7/14 NWs examined, not counting for β grains in contact with the catalyst which are common for most of the samples examined so far. We guess β-Sn grains are also present in other of the examined NWs but they were not visible due to the deposition orientation on the TEM grid. Regarding planar defects on the α-Sn phase, as discussed in the main text, we observe the majority of them in the {111} plane oblique to the NW axis, with very few exceptions, but twinning/stacking faults perpendicular to NW axis are notably shorter range than in the case of room temperature grown nanowires (some of these exceptions are visible in (b), middle). Scale bars are: top: 1 μm, middle & bottom: 10 nm.

S10. Additional HRTEM micrographs of 3nm Sn on InSb NW

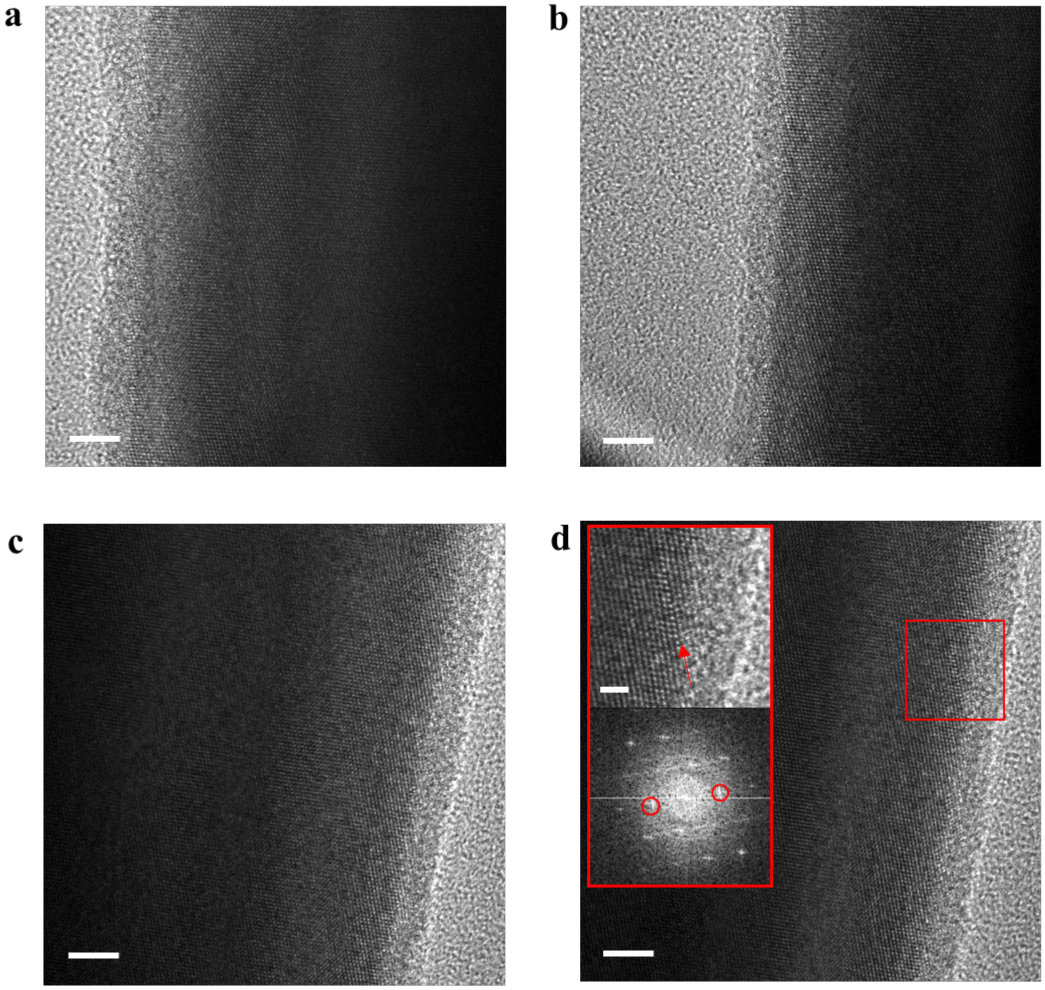

Figure S10: **a-d**, Details of Sn shell of 3 nm thickness. Shell forms a monocrystalline phase growing epitaxially on InSb. Only one initial stage twin formation is observed in (d) and it is oblique to the NW growth axis, as visualized in the inset FFT. Scale bars are: main: 5 nm, inset: 2 nm.

S11. Defect formation and initial β-Sn nucleation in 5 nm Sn shell on InSb NW

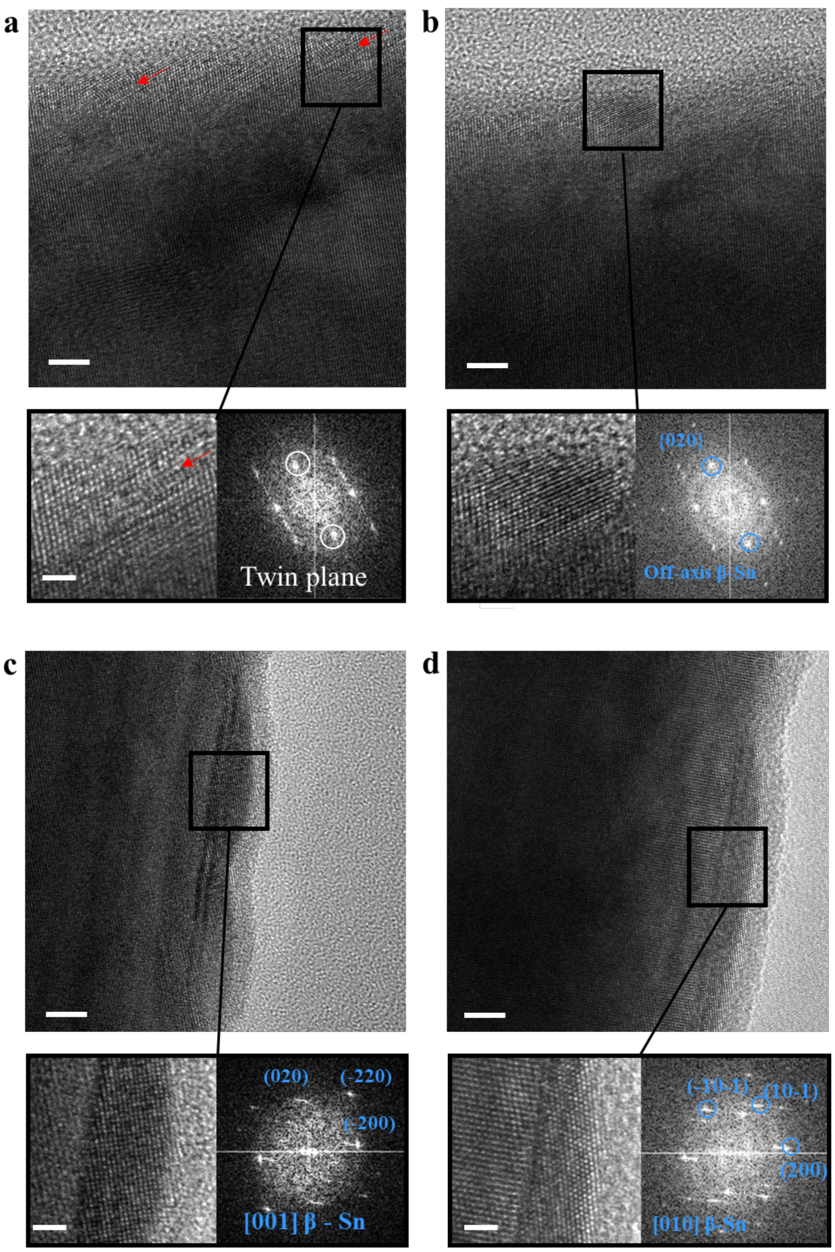

Figure S11: **a**, Details of initial stages of planar defect formation. Twinning events are indicated with red lines and the twinning plane oblique to NW growth axis is circled in the FFT. **b-d**, Detection of initial stages of β-Sn grains formation in different NWs. Scale bars are: main: 5 nm, zoom: 2 nm.

S12. Additional micrographs of 40nm Sn shell on InSb NW

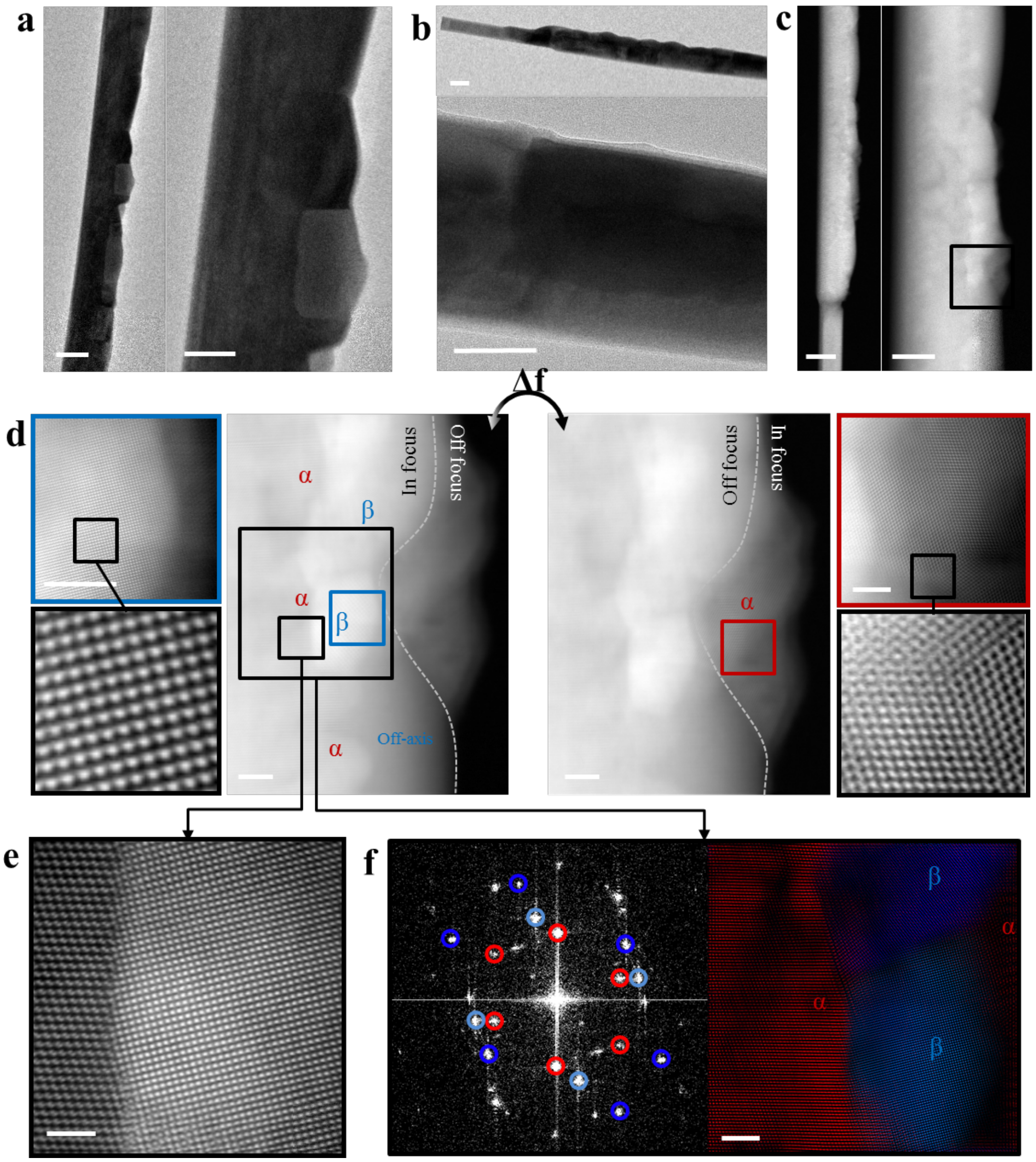

Figure S12: **a,b**, Low magnification HRTEM micrographs of different NWs showing a grainy shell. **c**, Low magnification HAADF micrographs of the NW shown in the main text. **d**, Zooming of the area marked in (c) imaged at different defocus. On the left, we observe several β-Sn grains growing on alpha. On the right, defective α-Sn phase is identified growing at different depth (different defocus) and therefore, not growing directly on top of β-Sn. **e**, Details on the interface between α and β -Sn. Matching between $\{200\}\beta$-Sn and $\{111\}\alpha$-Sn planes is observed by the appearance of quasi-periodic dangling $\{200\}\beta$ planes. **f**, Plane filtered map of this region highlighting the different spatial arrangement of Sn phases and grains. Scale bars are: (a) 100 nm & 100 nm, (b) 100 nm & 50 nm, (c) 100 nm & 50 nm, (d) 10 nm (inner panels), 5 nm (outer squared panels), (e) 2 nm, (f) 5 nm.

S13. Thermal stability of α-β-Sn/InSb heterostructure

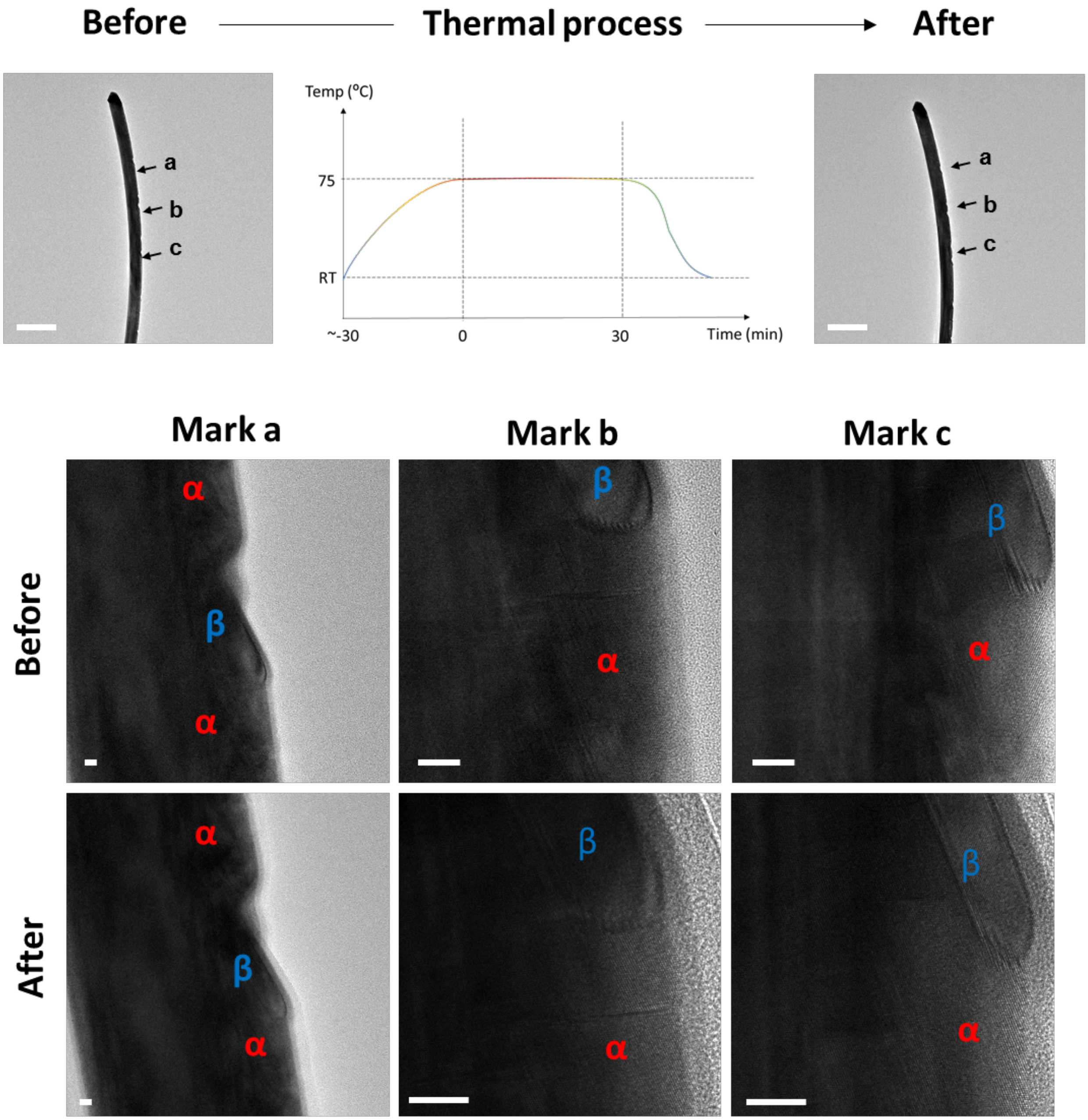

Figure S13: A heating process of 30 min at 75 ºC was applied to the InSb-Sn(20 nm) grown at -100 ºC in order to test the stability of the structure under temperature and try to promote β phase formation. After examining the same localized areas of the same nanowire before and after the process no structural variation was observed, neither β phase formation nor differences in α defective configurations. Scale bars are: top: 500 nm, bottom: 10 nm.

S14. Additional SEM images of InAsSb-Sn morphology

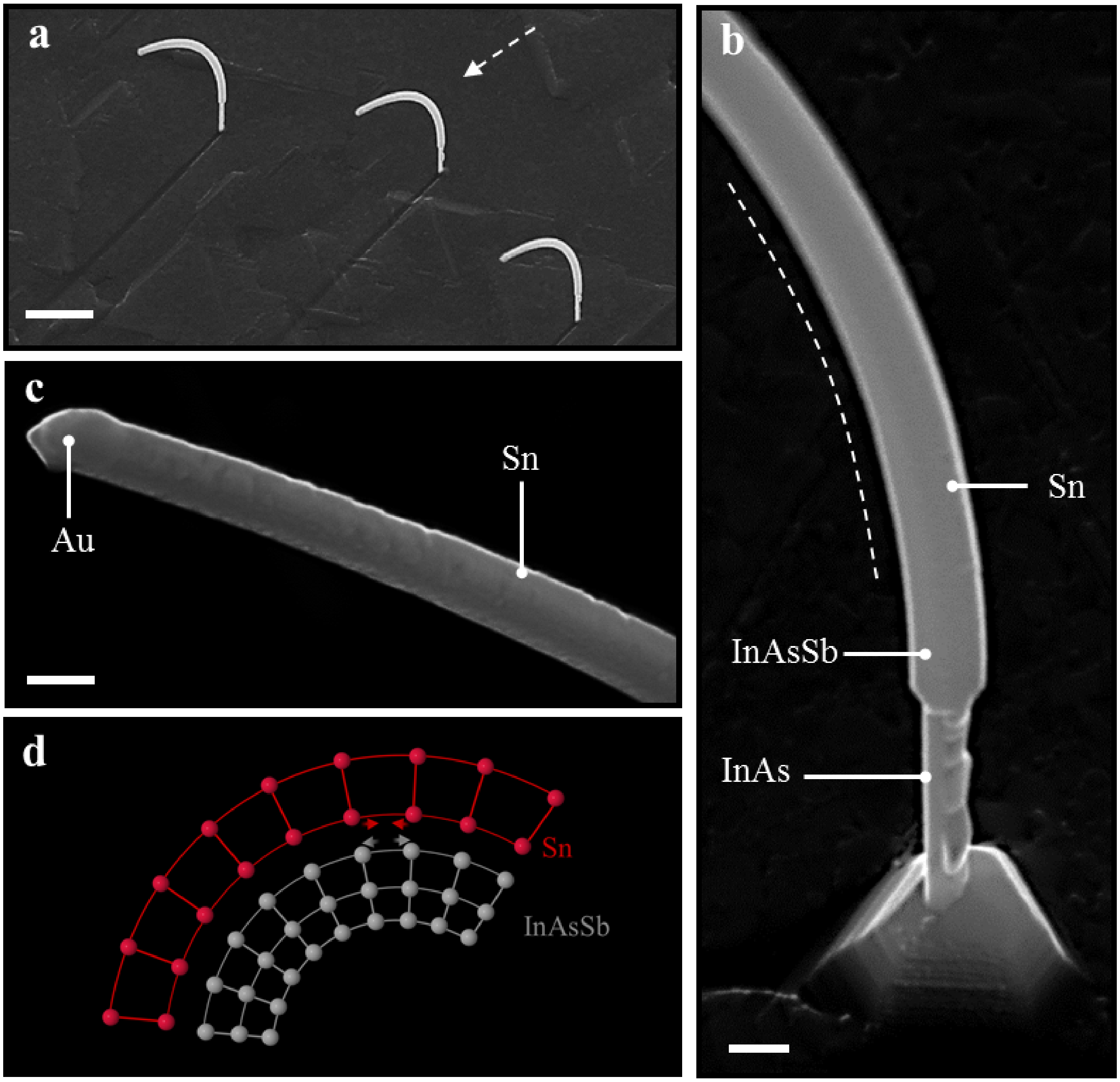

Figure S14: **a-c**, SEM images of Sn morphology on InAsSb NWs. As discussed in the main text, all the InAsSb-Sn hybrid shows strong bending away from the Sn deposition direction (arrow). On InAsSb, Sn exhibits rough morphology compare to InSb. At the top of NW (c), Sn film seems to be more inhomogeneous then the rest of the body (b). **d**, A schematic of atomic arrangement creating the strain-induced bending of the NWs. Note: the crystal orientation and lattice proportions do not fully reproduce the NW configuration, but act as a visual scheme. $\alpha-$Sn lattice gets compressive strained to match the InAsSb lattice constant and this lattice arrangement results in the bending of the NWs. Scale bars are: (a) 1μm, (b) and (c) 100 nm.

S15. Additional structural analysis of InAsSb/Sn

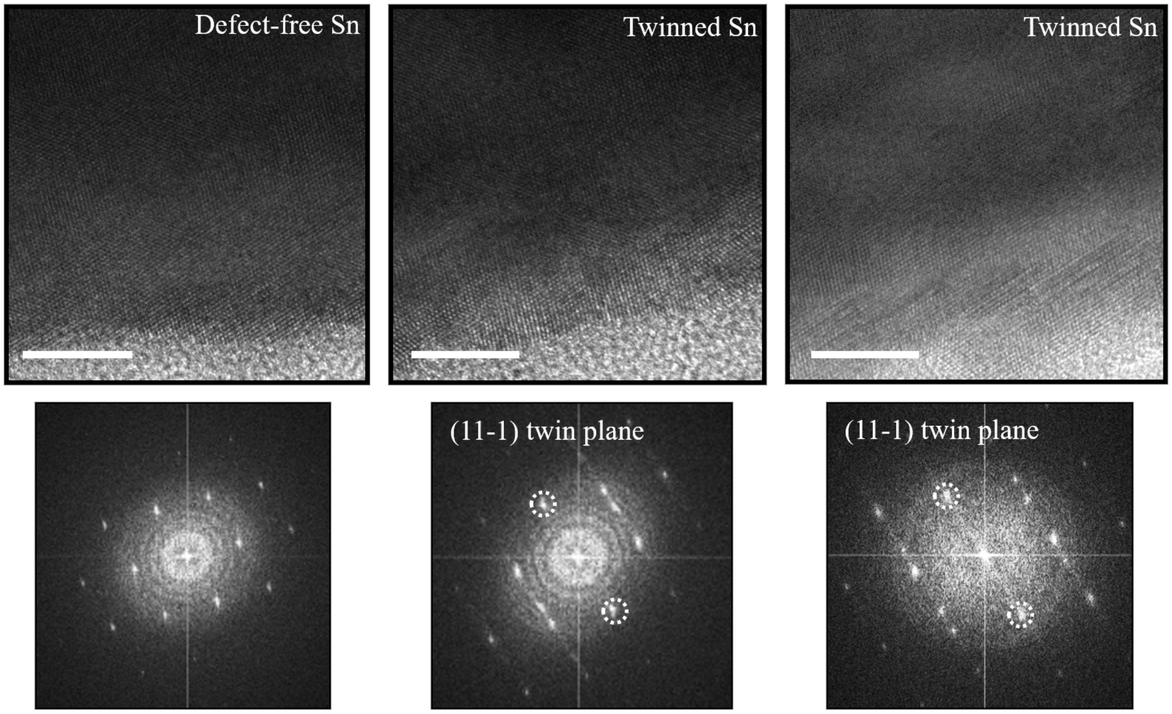

Figure S15: Additional representative micrographs of the main α-Sn configurations observed in InAsSb-Sn hybrid NWs. In the first of them, there are no twinning events, but defect-free α-Sn areas were rarely observed. In the middle and left figures, several planar defects (twin boundaries and stacking faults) are present in the structure forming $\sim 71°$ with the growth direction plane similar to InSb-Sn hybrids. Scale bars are 10 nm.

S16. Structural analysis of InAs/Sn NWs

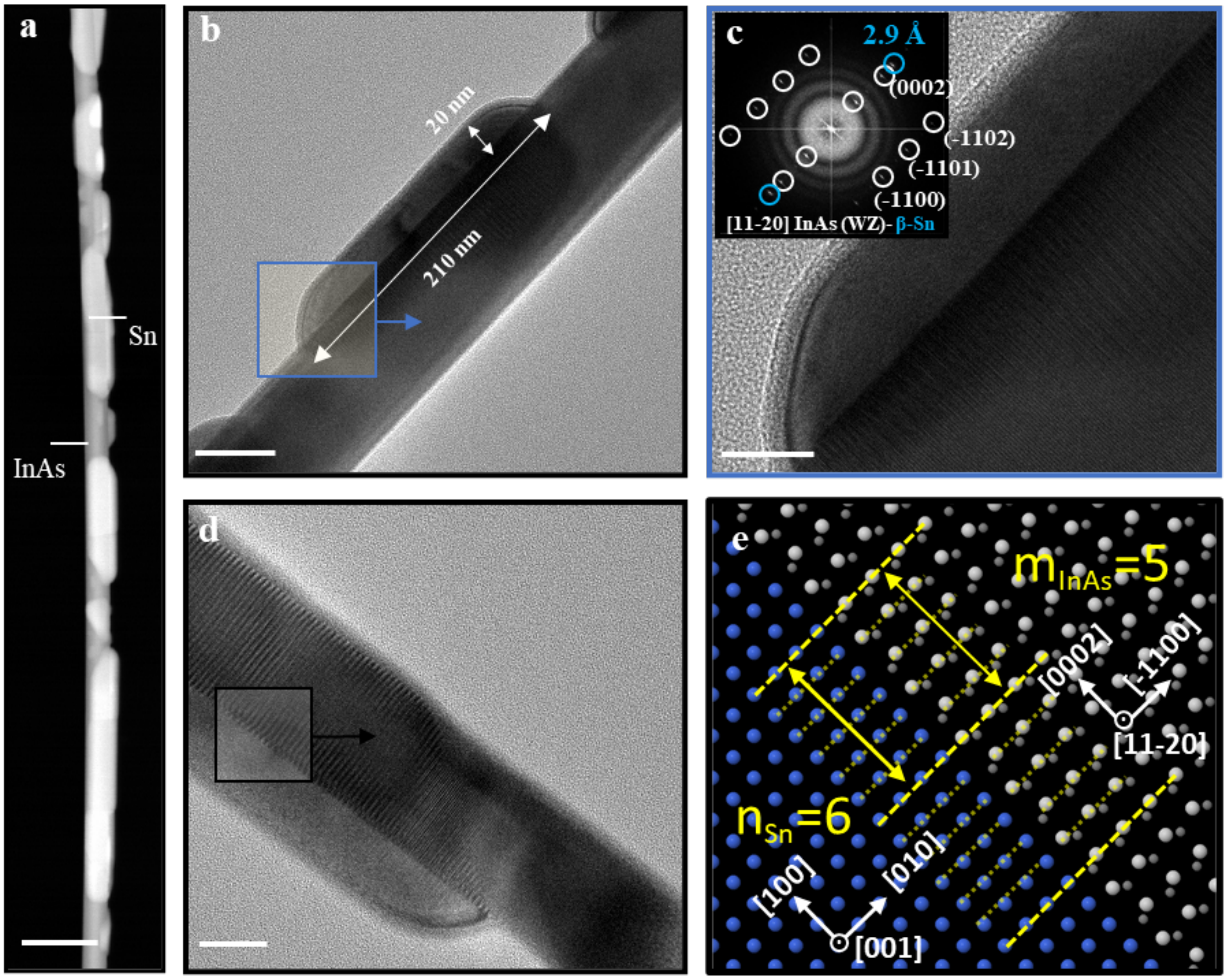

Figure S16: **a** HAADF-STEM micrograph of a InAs-Sn NW. In all cases discrete Sn grains of uniform ≈ 22 nm thickness and 100-600 nm width are observed. **b**, HRTEM micrograph of an InAs-Sn NW. **c**, Magnified micrograph of (b) with its corresponding indexed FFT. The FFT reveals that the Sn is disoriented with respect to InAs. However, the 2.9 Å Sn plane reflection is observed to be parallel to InAs(0002) **d**, HRTEM micrograph of a different NW where a Moirée pattern is visible on Sn-InAs overlap areas. **e**, Atomic Model of the Sn-InAs interface where Sn has been approached to [001] zone axis. A perfect domain creation of 6xSn(200):5xInAs(0002) can be observed. This domain creation matches the Moiré periodicity observed in the HAADF Micrographs. Scale bars are: (a) 200 nm, (b) 50 nm, (c) and (d) 20 nm.

S17. β-Sn phase analysis on InAs NW

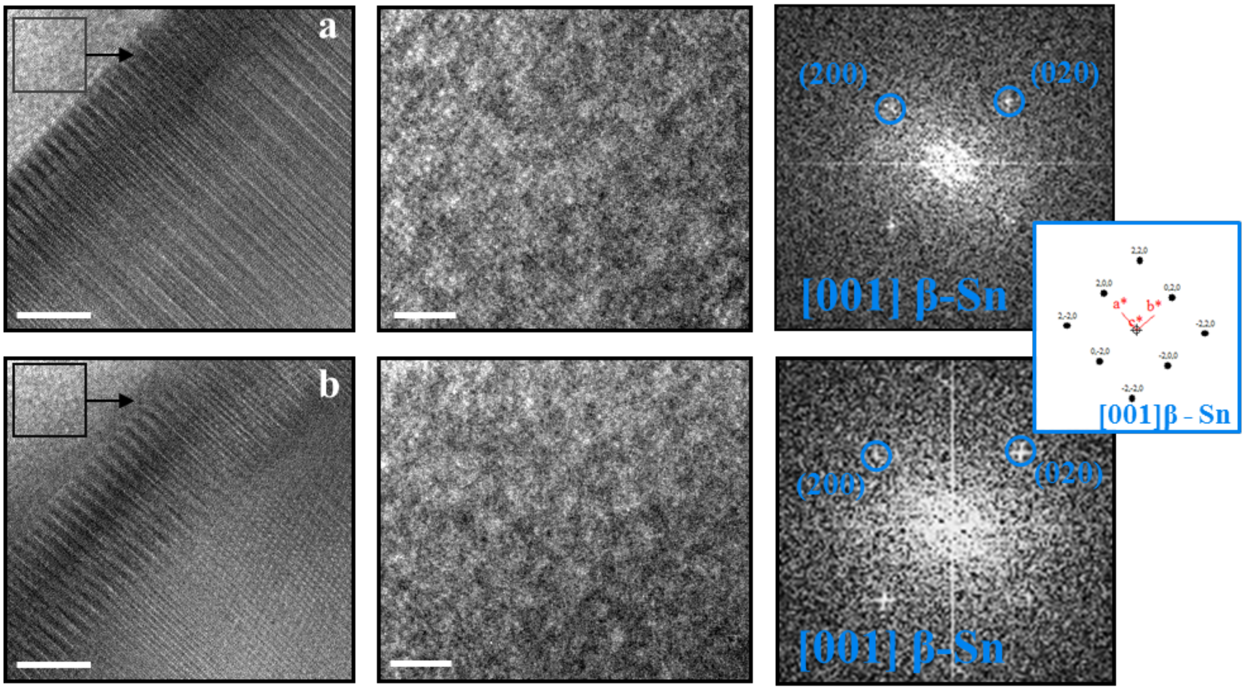

Figure S17: **a-b**, HRTEM micrographs of some other large grains whose orientation is close to [001] β-Sn zone axis. A dominant reflection of 2.9 Å is observed parallel to InAs(0002), as in the former section. This reflection matches β-Sn {200} planes. Additional reflection at 90 ° is also observed, meaning this crystal is close to [001] zone axis. Scale bars are 10 nm and 2 nm (for magnified images).

S18. Grain boundary within β-Sn on InAs facets

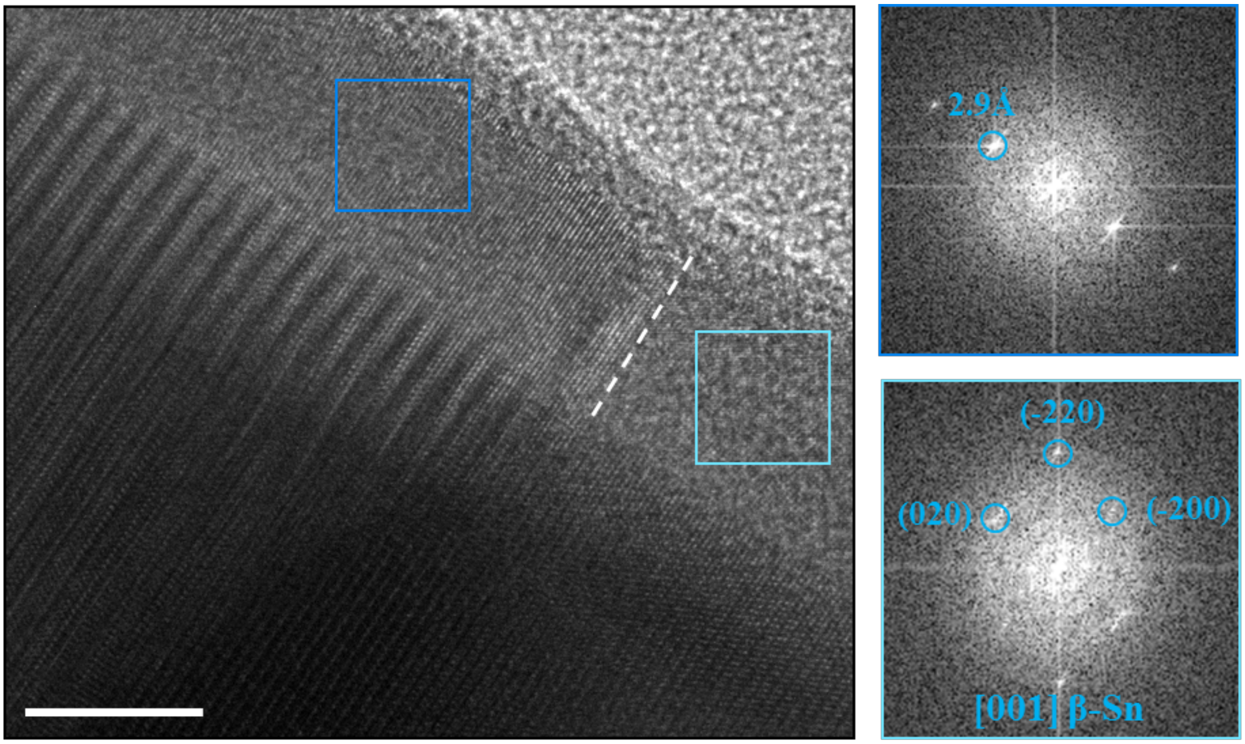

Figure S18: Some of the observed Sn islands on InAs present themselves more than one grain, probably originating from coalescence of smaller islands. This figure shows an example of two Sn crystals that are slightly disoriented between them, with their boundary position marked with white dashed lines. The top grain matches with disoriented β-Sn with visible {020} reflection parallel to InAs(0002). The bottom grain is identified as [001]- oriented β-Sn. Notice that despite the two grains are disoriented between them, they still keep the same in-plane longitudinal direction. Scale bar is 10 nm.

S19. Additional β-Sn Analysis on InAs NW

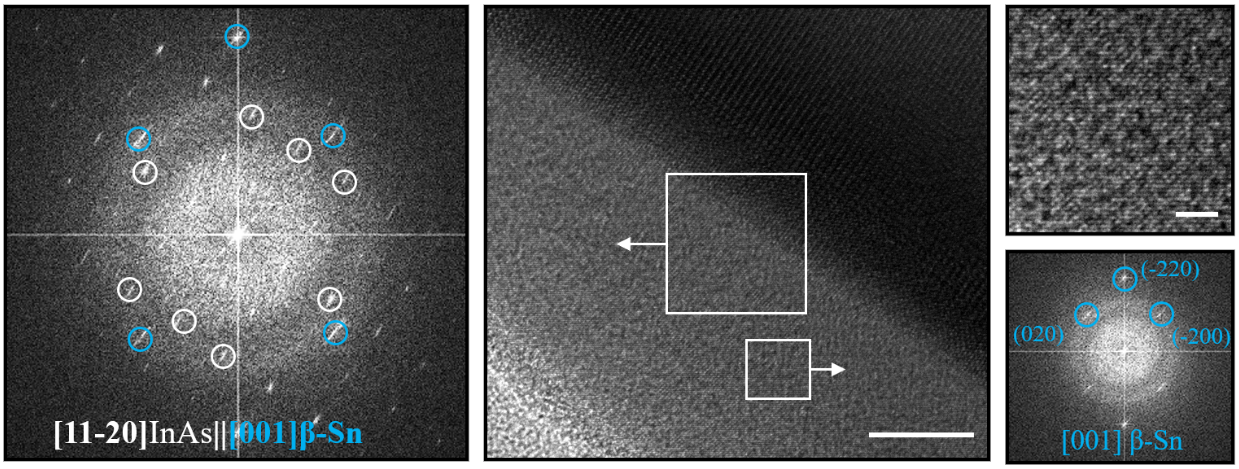

Figure S19: Additional HRTEM imaging and analysis in another InAs-Sn NW. β phase oriented along the [001] zone axis is observed. In this crystal, similar in-plane longitudinal plane match is observed, but Sn (020) plane is not strictly perpendicular to the interface. Scale bars are 10 nm and 2 nm.

S20. Additional β-Sn orientations on InAs NW

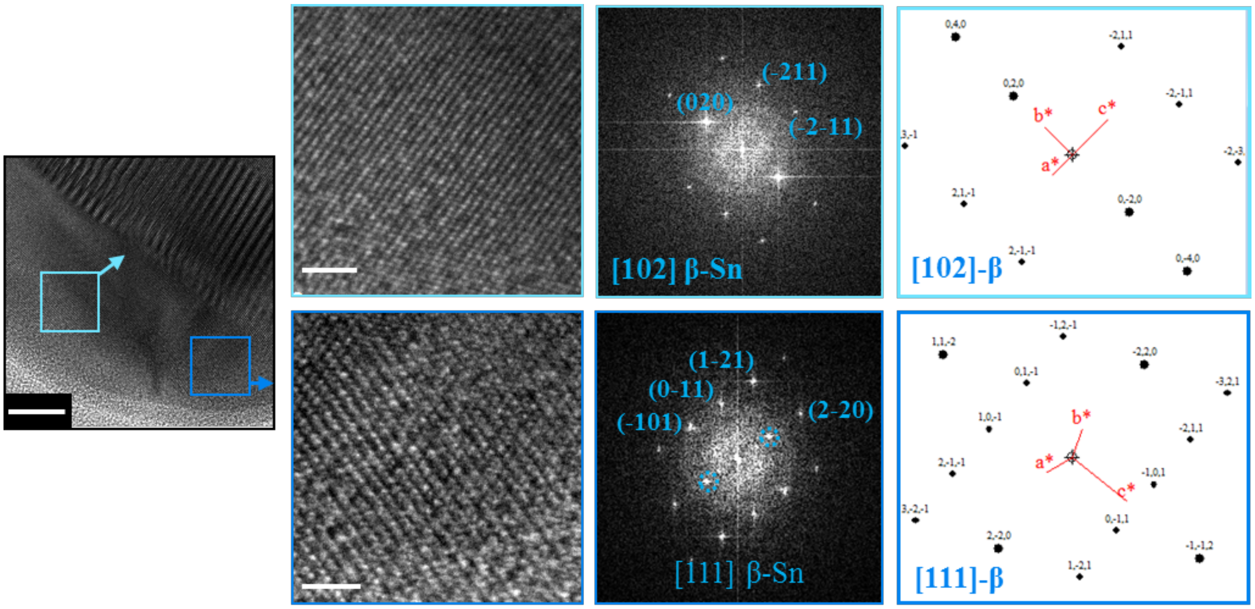

Figure S20: In this figure two other orientations of β-Sn are shown in a single island. We could identify a [102] β-Sn larger grain (top) which keeps same longitudinal in-plane configuration as the previously presented heterostructures. On the bottom, we could find a relatively small [111] oriented β-Sn grain that shows a completely different orientation with no similarities in configuration with larger grains (neither in-plane, nor out-of plane). Scale bars are 10 nm and 2 nm (for magnified images).

S21. InSb-InAs(2nm)-Sn

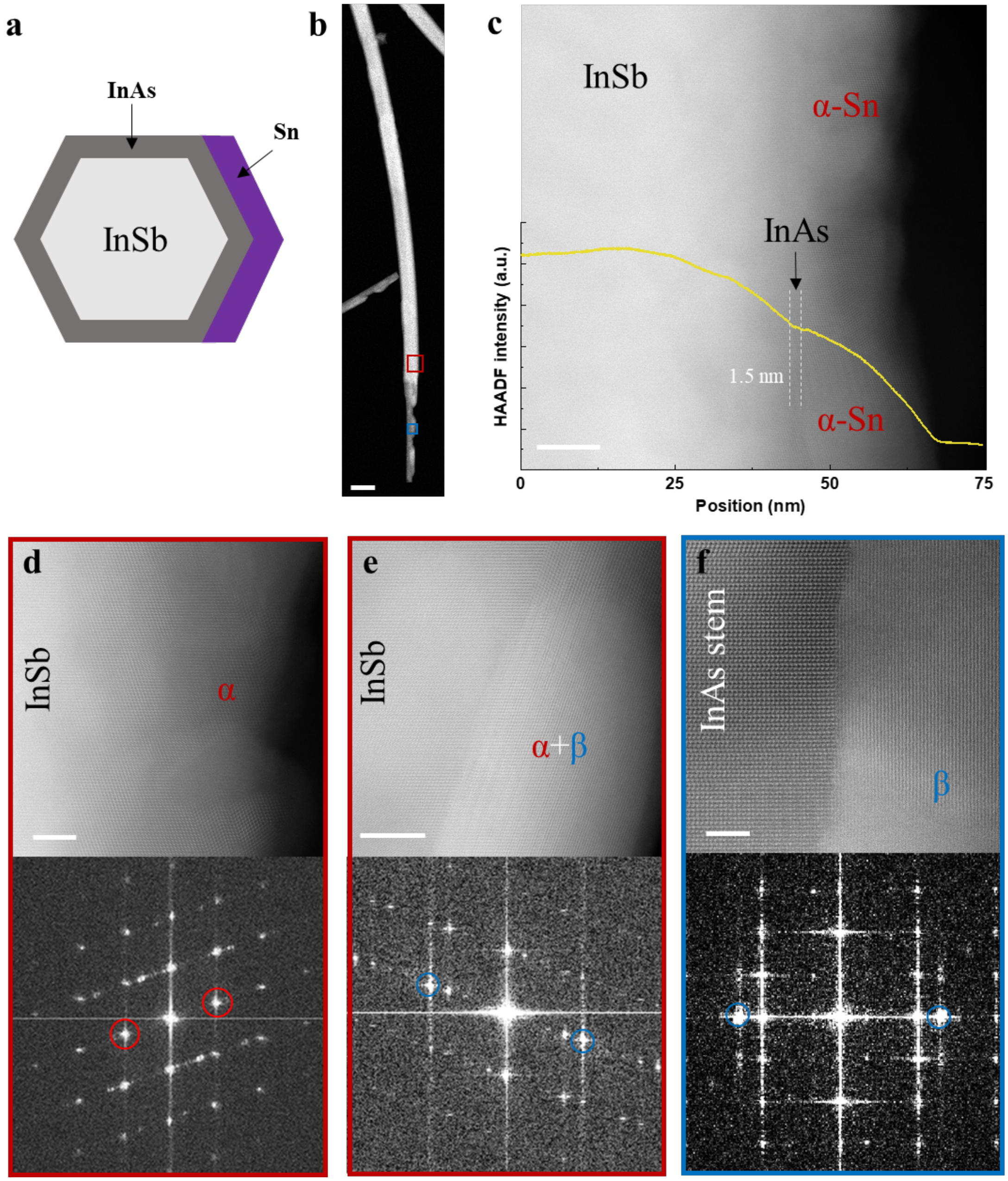

Figure S21: **a**, Cross-sectional schemetic of the NW configuration. **b**, Low-magnification HAADF micrograph of one InSb-InAs-Sn NW. **c**, HAADF details on the shell. The InAs shell is distinguished by the presence of a slight decrease in HAADF intensity. The majority of the shell on the InSb section of the NW belongs to α-Sn with oblique twinning defects, as observed in pure InSb-Sn NWs. **d,e**, Close up view to the shell on the InSb region and FFTs. Red circles indicate the twinning plane and blue circles β-Sn planes in overlap with α. **f**, β phase identified on the InAs stem. Scale bars are: (b) 200 nm, (c) 10 nm, (d-f) 5 nm.

S22. Summary of 3D models of the α-β/InX configuration

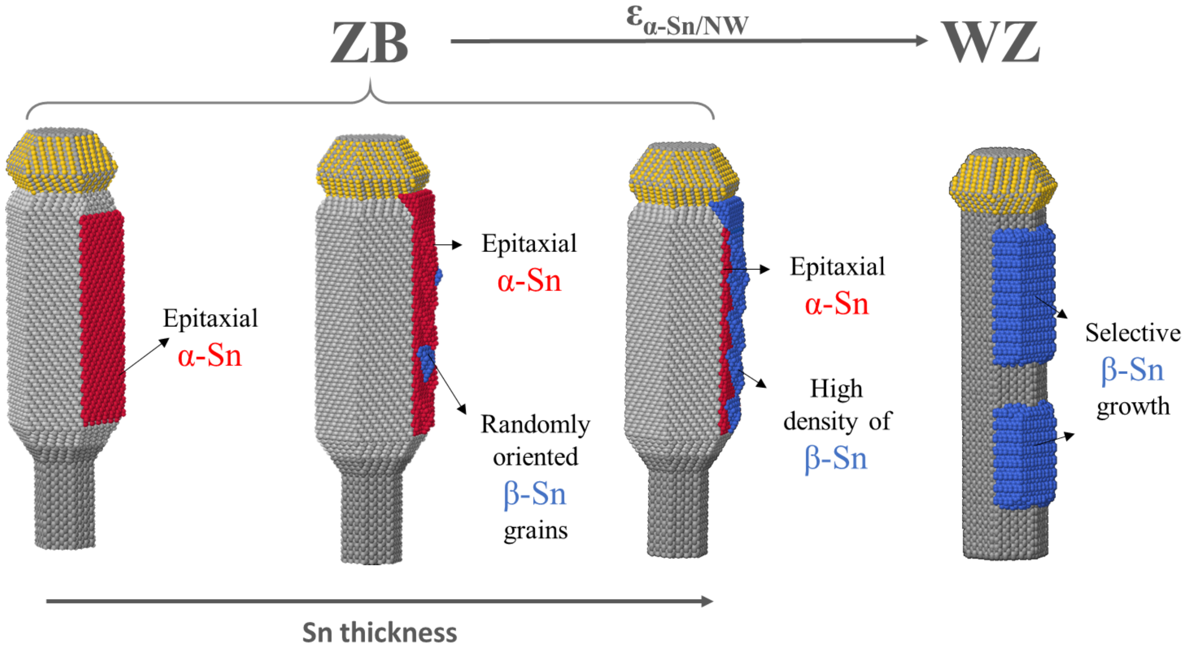

Figure S22: Schematics of Sn phase formation depending on its thickness and crystal structure of the template nanowires.

S23. Growth parameters of the fabricated devices

Table S1: Growth conditions of the hybrid NW-Sn devices presented in the main text.

#	**NW material**	**Holder Temp (ºC)**	**Sn Thikness (nm)**
D1	InSb-InAs (2 nm)	≈ -150	≈ 15
D2	InSb	-50	≈ 20
D3	InSb	RT	≈ 20
D4	InSb	-150	≈ 15
D5	InAsSb	-150	≈ 15
D6	InAsSb	-150	≈ 15
D7	InSb	-150	≈ 15
D8	InSb	-150	≈ 15
D9	InSb	-150	≈ 15

S24. Additional Device Measurement

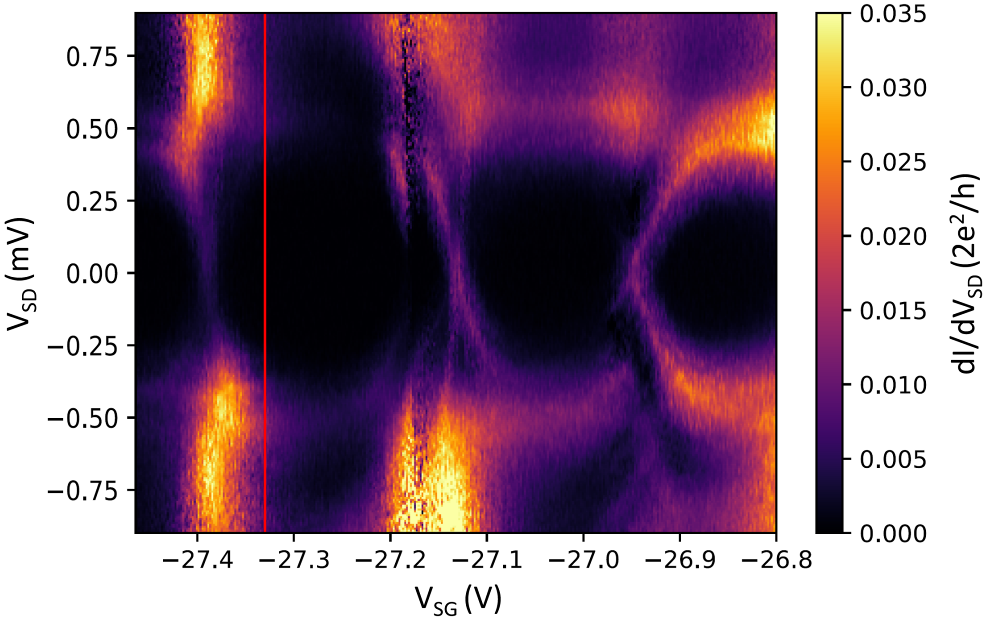

Figure S23: Differential conductance dI/dV_{SD} as a function of source-drain voltage V_{SD} and side-gate voltage V_{SG} for the device depicted in Fig. 5g of the main text. Negative V_{SG} caused depletion of electrons in the bare InAs nanowire segment, until the device reached a regime of Coulomb blockade. Here, the InAs nanowire segment acts as a tunnel barrier, and dI/dV_{SD} becomes proportional to the density of states in the hybrid. The horizontal, V_{SD}-independent lines arise from gap $\Delta \sim 0.44$ meV in the superconducting density of states. Additional sub-gap features are related to the hybridisation between the superconductor and semiconductor.[12–14] The red vertical line shows the V_{SG} for which the data in main text Figure 5f was collected.

References

(1) Swanson, H. E.; Fuyat, R. K. Standard X-ray diffraction powder patterns. National Bureau of Standards Circular (U. S.) **1953**, 539, 1–65.

(2) Wolcyrz, M.; Kubiak, R.; Maciejewski, S. X-ray investigation of thermal expansion and atomic thermal vibrations of tin, indium, and their alloys. Physica Status Solidi B: Basic Solid State Physics **1981**, 107, 245–253.

(3) Vegard, L. Die Konstitution der Mischkristalle und die Raumfüllung der Atome. Zeitschrift für Physik **1921**, 5, 17–26.

(4) Spirkoska, D. et al. Structural and optical properties of high quality zinc-blende/wurtzite GaAs nanowire heterostructures. Phys. Rev. B **2009**, 80, 245325.

(5) Arbiol, J.; Fontcuberta i Morral, A.; Estradé, S.; Peiró, F.; Kalache, B.; Roca i Cabarrocas, P.; Morante, J. R. Influence of the (111) twinning on the formation of diamond cubic/diamond hexagonal heterostructures in Cu-catalyzed Si nanowires. Journal of Applied Physics **2008**, 104, 064312.

(6) Khan, S. A.; Lampadaris, C.; Cui, A.; Stampfer, L.; Liu, Y.; Pauka, S. J.; Cachaza, M. E.; Fiordaliso, E. M.; Kang, J.-H.; Korneychuk, S., et al. Highly transparent gatable superconducting shadow junctions. ACS nano **2020**, 14, 14605–14615.

(7) Krogstrup, P.; Ziino, N.; Chang, W.; Albrecht, S.; Madsen, M.; Johnson, E.; Nygård, J.; Marcus, C.; Jespersen, T. Epitaxy of semiconductor–superconductor nanowires. Nature Materials **2015**, 14, 400–406.

(8) Thompson, C.; Floro, J.; Smith, H. I. Epitaxial grain growth in thin metal films. Journal of applied physics **1990**, 67, 4099–4104.

(9) Farrow, R.; Robertson, D.; Williams, G.; Cullis, A.; Jones, G.; Young, I.; Dennis, P.

The growth of metastable, heteroepitaxial films of α-Sn by metal beam epitaxy. Journal of Crystal Growth **1981**, 54, 507–518.

(10) Höchst, H.; Calderón, I. H. Microscopic electronic structure and growth mode of Sn/InSb (111) interfaces. Journal of Vacuum Science & Technology A: Vacuum, Surfaces, and Films **1985**, 3, 911–914.

(11) Magnano, E.; Pivetta, M.; Sancrotti, M.; Casalis, L.; Fantini, P.; Betti, M. G.; Mariani, C. Growth morphology and electronic properties of Sn deposited on different InSb surfaces. Surface science **1999**, 433, 387–391.

(12) Chang, W.; Albrecht, S.; Jespersen, T.; Kuemmeth, F.; Krogstrup, P.; Nygård, J.; Marcus, C. Hard gap in epitaxial semiconductor–superconductor nanowires. Nature Nanotechnology **2015**, 10, 232–236.

(13) Mourik, V.; Zuo, K.; Frolov, S. M.; Plissard, S. R.; Bakkers, E. P. A. M.; Kouwenhoven, L. P. Signatures of Majorana Fermions in Hybrid Superconductor-Semiconductor Nanowire Devices. Science **2012**, 336, 1003–1007.

(14) Lee, E. J.; Jiang, X.; Houzet, M.; Aguado, R.; Lieber, C. M.; De Franceschi, S. Spin-resolved Andreev levels and parity crossings in hybrid superconductor–semiconductor nanostructures. Nature Nanotechnology **2014**, 9, 79.
